# 3D Bioprinted Coaxial Testis Model Using Human Induced Pluripotent Stem Cells:A Step Toward Bicompartmental Cytoarchitecture and Functionalization

**DOI:** 10.1002/adhm.202402606

**Published:** 2025-02-16

**Authors:** Meghan A Robinson, Sonia HY Kung, Khaled YM Youssef, Kali M Scheck, Robert H Bell, Funda Sar, Anne M Haegert, M Mahdi Asmae, Changfeng Cheng, Salina V Yeack, Bhairvi T Mathur, Feng Jiang, Colin C Collins, Faraz Hach, Stephanie M Willerth, Ryan K Flannigan

**Affiliations:** ^1^ Vancouver Prostate Centre Vancouver British Columbia V6H 3Z6 Canada; ^2^ Starfish Medical Toronto Ontario M6N 3C5 Canada; ^3^ Axolotl Biosciences Victoria British Columbia V8W 2Y2 Canada; ^4^ Faculty of Forestry University of British Columbia Vancouver British Columbia V6T 1Z4 Canada; ^5^ Faculty of Medicine University of British Columbia Vancouver British Columbia V6T 1Z4 Canada; ^6^ Department of Mechanical Engineering University of Victoria Victoria British Columbia V8P 5C2 Canada; ^7^ Division of Medical Sciences University of Victoria Victoria British Columbia V8P 5C2 Canada; ^8^ Department of Urologic Sciences University of British Columbia Vancouver British Columbia V6T 1Z4 Canada

**Keywords:** bioprinting, human induced pluripotent stem cell, in vitro spermatogenesis, microsphere delivery, single cell RNA sequencing

## Abstract

Fertility preservation following pediatric cancer therapy programs has become a major avenue of infertility research. In vitro spermatogenesis (IVS) aims to generate sperm from banked prepubertal testicular tissues in a lab setting using specialized culture conditions. While successful using rodent tissues, progress with human tissues is limited by the scarcity of human prepubertal testicular tissues for research. This study posits that human induced pluripotent stem cells (hiPSCs) can model human prepubertal testicular tissue to facilitate the development of human IVS conditions. Testicular cells derived from hiPSCs are characterized for phenotype markers and profiled transcriptionally. HiPSC‐derived testicular cells are bioprinted into core–shell constructs representative of testis cytoarchitecture and found to capture functional aspects of prepubertal testicular tissues within 7 days under xeno‐free conditions. Moreover, hiPSC‐derived Sertoli cells illustrate the capacity to mature under pubertal‐like conditions. The utility of the model is tested by comparing 2 methods of supplementing retinoic acid (RA), the vitamin responsible for inducing spermatogenesis. The model reveals a significant gain in activity under microsphere‐released RA compared to RA medium supplementation, indicating that the fragility of free RA in vitro may be a contributing factor to the molecular dysfunction observed in human IVS studies to date.

## Graphical abstract

The ability of human induced pluripotent stem cells (hiPSCs) to model developmental processes makes them a promising source for engineering testicular tissues to study in vitro maturation and sperm production. This work describes the first hiPSC‐derived testicular model and shows that it captures functional aspects of prepubertal testicular tissues within 7 days.

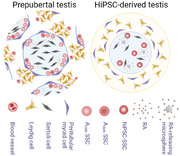

## 1 Introduction

Fertility preservation is an important quality of life consideration following pediatric cancer therapy programs. The rate of survival after pediatric cancer has risen to over 80%; however, due to the gonadotoxic nature of cancer therapies, 42–66% of male survivors are expected to suffer from infertility as adults.^[^ ^1^ ^]^ To date, fertility preservation means offering pre‐ and peri‐pubertal patients the option to bank fragments of testis tissue containing spermatogonial stem cells (SSCs) prior to gonadotoxic cancer treatments. The theory underlying this practice is that SSCs have the potential to generate sperm for use later in life in assisted reproductive therapies. A recent report estimates 989 patients have banked SSCs to date, with some, now adults, pursuing parenthood.^[^ ^2^ ^]^ Meanwhile, technologies to differentiate banked SSCs into sperm remain in the research phase.^[^ ^3^ ^]^

Using non‐human primate models, researchers have demonstrated that autologous transplant of prepubertal SSCs back into the patient post‐puberty can regenerate sperm and healthy offspring.^[^ ^4^ ^]^ However, translating this technique to humans is ultimately challenged by the risk of reintroducing malignant cancer cells.^[^ ^5^ ^]^ Evidence from rodent studies illustrates that as few as 20 leukemic cells from an SSC transplant can cause a cancer relapse.^[^ ^6^ ^]^ In light of this, researchers are also developing a safer approach termed in vitro spermatogenesis (IVS). IVS circumvents risk to patients by transferring the sperm differentiation process to a lab setting. This method was first successful using mouse testicular tissue, and recently, with adjustments proved successful with rat testicular tissue.^[^ ^7^ ^]^ Recent work aims to adapt this method using porcine testicular tissue and human testicular tissue.^[^ ^8^ ^]^ Human studies show promise, with germ cell differentiation up to the spermatid stage in some cases, but nearly all studies report degeneration of the tissue and SSC loss over time.^[^ ^3^ ^]^ Progress is slow as efforts are restricted by difficulty in sourcing human prepubertal testis tissues for research.^[^ ^9^ ^]^ The scope of human IVS research to date is limited to a handful of studies, in which analysis and statistical power are likewise limited by small amounts of tissue.

We propose that a prepubertal human testis tissue model generated from human induced pluripotent stem cells (hiPSCs) could advance IVS research by bridging the gap between animal models and rare human prepubertal patient tissues. HiPSCs are created from somatic cells such as fibroblasts or blood cells by over‐expressing a set of transcription factor genes responsible for pluripotency. Like human embryonic stem cells (hESCs), they divide indefinitely and differentiate into any cell type in the body, representing a renewable resource for human tissue modeling.^[^ ^10^ ^]^ Furthermore, similar to animal models, their regenerative nature opens the door to genetically engineered models incorporating gene knock‐outs or reporters for interrogation and real‐time visualization of key molecular drivers.^[^ ^11^ ^]^

This study describes a 3‐dimensional (3‐D) bicompartmental hiPSC‐derived testis tissue model. Testicular cells are first derived from hiPSCs and characterized for their phenotype and transcriptomic profiles using immunostaining and single‐cell sequencing and then bioprinted into core‐shell fibers representing the interstitial and tubular compartments of testis cytoarchitecture. This study characterizes the activity of the core‐shell cytoarchitecture in comparison to a core‐only cytoarchitecture, revealing cross‐talk between shell and core, characteristic of a prepubertal state. It further shows that under conditions mimicking the pubertal environment the model has the capacity to mature. Finally, we use the model to interrogate the utility of retinoic acid‐releasing microspheres (RA‐MS) over standard retinoic acid (RA) supplementation to support RA‐mediated testicular function in an in vitro setting where RA is known to be rapidly degraded by light, heat, and oxygen.^[^ ^12^ ^]^

## 2 Experimental Section

### 2.1 Ethical Approval

Ethical approval for the use of human tissue was obtained through the Clinical Research Ethics Board (CREB)‐approved protocols H18‐03543 and H17‐02860. The primary human testis single‐cell sequencing datasets used were public. Experiments using hiPSCs in this study were not subject to ethics approval from CREB or the Stem Cell Oversight Committee (SCOC), since they were derived from somatic cells and not intended for transfer into humans or non‐human animals.

### 2.2 hiPSC Culture

HiPSC lines used were iPS(Foreskin) ‐1 (WiCell, lot DL01) and iPS11‐10 (Cell Applications). HiPSCs were expanded on Growth Factor Reduced Matrigel (Corning, 354 230) in mTeSR Plus medium (Stemcell Technologies, 100‐0276) and passaged using ReLeSR enzyme‐free selective passaging reagent (Stemcell Technologies, 05872).

### 2.3 Differentiation and Culture of Testis Cells from hiPSCs

SSCs, Leydig cells, peritubular myoid cells, and Sertoli cells were differentiated from hiPSCs as previously described.^[^ ^13^ ^]^ Following differentiation, Leydig cells were expanded on hESC‐Qualified Growth Factor Reduced Geltrex (Gibco, A1569601) in Leydig Cell Medium (Sciencell, 4511) supplemented with 10 ng mL^−1^ Leukemia Inhibiting Factor (LIF, Peprotech, AF‐300‐05), 10 ng mL^−1^ Epidermal Growth Factor (EGF, Peprotech, AF‐100‐15), 10 ng mL^−1^ Vascular Endothelial Growth Factor (VEGF) ‐165 (Peprotech, 100‐20), 10 ng mL^−1^ Luteinizing Hormone (LH, Millipore Sigma, L6420) and 10 ng mL^−1^ heat stable Fibroblast Growth Factor 2 (FGF2, Thermofisher, PHG0367). Expansion of Leydig cells under these conditions was limited to ≈3 passages. Peritubular myoid cells were expanded on tissue culture‐treated plastic in Dulbecco's Modified Eagle Medium/Nutrient Mixture F‐12 (DMEM/F12) with 10% Fetal Bovine Serum (FBS, Gibco, 10 437 028). Sertoli cells were expanded on tissue culture‐treated plastic in Sertoli Cell Medium (Sciencell, 4521). SSCs were expanded on a sulfated dextran‐4‐armed polyethylene glycol (starPEG) hydrogel matrix functionalized with vitronectin and fibronectin type II domain (FN2) peptide motifs (denovoMATRIX, GmbH) in Human Plasma‐Like Medium (HPLM), 15% Cell Therapy Systems (CTS) KnockOut Serum Replacement (SR) XenoFree supplement (Gibco, 12 618 012), 1 µM vitamin C (Millipore Sigma, A4403), 1 µM vitamin E (Millipore Sigma, T1157), 10 µg mL^−1^ biotin (Millipore Sigma, B4639), 60 ng mL^−1^ progesterone (Millipore Sigma, P8783), 60 µM putrescine (Millipore Sigma, P5780), 30 µg mL^−1^ pyruvate (Millipore Sigma, S8636), 30 ng mL^−1^ β‐estradiol (Sigma, E2758), 1X Minimum Essential Medium (MEM) Vitamin Solution (Gibco, 11 120 052), 1X GlutaMAX Supplement (Gibco, 35 050 061), 1 µL mL^−1^ DL‐lactate (Millipore Sigma, L4263), 50 µM β‐mercaptoethanol (Gibco, 31350‐010), 15 ng mL^−1^ Glial Cell‐Derived Neurotrophic Factor (GDNF, Peprotech, AF‐450‐010), 10 ng mL^−1^ heat stable FGF2, and either 10 ng mL^−1^ EGF or 100 nM Akt pathway inhibitor MK2206 (Biogems, 1 031 320). All were cryopreserved in CryoStor CS10 Freezing Medium (Stemcell Technologies, 07 930). Thawing of hiPSC‐Leydig and hiPSC‐SSCs required 24 h addition of the rho‐associated protein kinase (ROCK) inhibitor 1X RevitaCell Supplement (Gibco, A2644501).

### 2.4 RA Microsphere Generation

RA was encapsulated using a microfluidic device (see Supporting Information for the design of the device). The aqueous phase was prepared as 2% w/v Poly(vinyl alcohol) (PVA, MW 13 000–23 000, 87–89% hydrolyzed, Millipore Sigma, 363 170) in deionized (DI) ‐water. Briefly, 8 g of PVA in 400 mL of DI‐water were stirred at 850 rpm at 85 °C until a clear solution was formed. The oil phase (polymer phase) was prepared using polycaprolactone (PCL, MW 80 000, Millipore Sigma, 440 744) dissolved in dichloromethane (DCM, Millipore Sigma, DX0838) at a concentration of 5% w/v. 0.8 g of PCL was mixed with 15 mL of DCM in a tight container at 900 rpm. RA (Millipore Sigma, R2625) was dissolved in 100% ethanol and mixed with the oil phase to a final concentration of 6 µg mg^−1^ (w/w RA/PCL). A pressure‐based delivery system consisting of an air compressor, a pressure regulator, and a flow rate sensor (Fluigent) was used for the aqueous phase delivery. A syringe pump was used for the dispersed phase. Initially, the aqueous phase was introduced to purge the system from the air in the tubing and wet the device for 2 min at 5 mL min^−1^. Then the dispersed phase was introduced using the syringe pump at 1 mL min^−1^. Within 2 min a stable flow and consistent droplet formation were observed. The produced droplets were filtered using an inline 25 µm filter (McMaster‐Carr, 4795K21) connected at the outlet side in a container filled with 2% PVA solution. After collection, the solution was stirred at 39 °C and 850 rpm for 4 h to extract DCM.

Encapsulation efficiency was determined using spectrophotometry. Briefly, 10 mg of RA‐MS were added to 100 µL of DCM and vortexed until they were completely dissolved. 1 mL of 100% ethanol was then added to the solution and mixed to allow the DCM to evaporate and precipitate out the PCL. The samples were then centrifuged at 6000 rpm for 10 s. The supernatant was analyzed using a Nanodrop 2000 Spectrophotometer (Thermo Scientific) at a wavelength of 354 nm. This was done in triplicate and represented as the mean with standard deviation.

Size and uniformity were determined by dynamic light scattering (DLS). Lyophilized microspheres were resuspended in 100% ethanol at a concentration of 0.1 mg mL^−1^ and transferred to a 10 mm × 100 mm quartz cuvette (Malvern Panalytical, PCS8501). The sample was analyzed in quadruplicate using a Zetasizer, (Malvern Panalytical) and the software used was ZS Explorer (Malvern Panalytical). The material was set as polystyrene latex, and the dispersant was ethanol. The equilibration time was set at 120 s, and the angle of detection was back scatter.

An RA release profile was generated by incubating RA‐MS in PBS at 34 °C and 5% CO_2_ every 48 h over 10 days and measuring the light absorbance at 354 nm using an Infinite 200 Pro microplate reader (Tecan). The solution collected every 48 h, was centrifuged at 1000 rpm for 5 min to precipitate the microspheres, and the supernatant was analyzed for its light absorbance. The microsphere pellet was resuspended in fresh phosphate buffered saline (PBS) and placed back in the incubator every 48 h to measure fresh release. The sampling and measurements were done in triplicate and reported as the mean absorbance with standard deviation.

### 2.5 Rheological Characterization of the Bioinks

The rheological properties of the bioinks were measured at room temperature by a rheometer (AR2000, TA Instruments) using a cone plate configuration with a diameter of 25 mm and a gap of 1000 µm. The continuous ramp viscosity test was carried out at the shear rate from 0.01 to 100 s^−1^. The stress and strain sweep tests were carried out at 1–1000 Pa of oscillatory stress with an angular frequency of 6.283 rad s^−1^ at room temperature.

### 2.6 Bioprinting

Core‐shell constructs were made with a BioX^6^ extrusion bioprinter (Cellink) fitted with a 22‐18 gauge coaxial needle (Rame‐Hart, 100‐10‐COAXIAL‐2218). HiPSC‐derived Sertoli cells and SSCs were suspended in the core bioink while hiPSC‐derived Leydig and peritubular myoid cells were suspended in the shell bioink. Printing was done at a speed of 1 mm s^−1^ with shell pressures between 240‐280 kPa and core pressure of 70 kPa. Each print was 3–4 cm long and printed in a 1 × 1 cm square configuration to improve stability. Constructs without shells were created by setting the shell pressure to 0 kPa. Some constructs were immediately analyzed as day 0 controls, and the rest were placed in 2 mL medium and cultured at 34 °C, 5% CO_2_, and 21% O_2_ for 7 days. The xeno‐free medium formulation was designed to mimic human plasma with key endocrine factors. Its composition was HPLM, 15% CTS KnockOut SR XenoFree supplement, 10 ng mL^−1^ triiodothyronine (T3, Millipore Sigma, T6397), 10 ng mL^−1^ LH, 10 ng mL^−1^ follicle stimulating hormone (FSH, Millipore Sigma, F4021), 1 µM testosterone (Toronto Research Chemicals, T155010), 60 µM putrescine (Millipore Sigma, P5780), 10 ng mL^−1^ 22‐hydroxycholesterol (Millipore Sigma, H9384), and 1 µM retinol (Millipore Sigma R7632). The bioink used for the core and shell components were VitroINK YIGSR (TheWell Bioscience, INK05‐3) and VitroINK COL (TheWell Bioscience, INK01‐3), respectively.

For the RA‐MS experiments, since previously published IVS experiments report a concentration range of 0.3‐10 µM, core‐shells were printed with or without 0.05 mg mL^−1^ RA‐MS mixed into the core bioink to amount to 1 µM RA cumulative total release, while those without RA‐MS were supplemented with 10 µM RA by adding it to their medium.^[^ ^3^ ^]^ The xeno‐free medium formulation was slightly modified in an effort to improve growth. Its composition was HPLM, 15% CTS KnockOut SR XenoFree supplement, 10 ng mL^−1^ LH, 10 ng mL^−1^ FSH, 1 µM testosterone, 60 µM putrescine, 10 ng mL^−1^ 22‐hydroxycholesterol, 1 µM retinol, 1 µM vitamin C, 1 µM vitamin E, 10 ng mL^−1^ biotin, and 1X Insulin‐Transferrin‐Selenium (ITS) supplement (Gibco, 41 400 045). In one condition, Bone Morphogenic Protein 4 (BMP4, Peprotech 120‐05ET) and KIT Ligand/Stem Cell Factor (KITLG/SCF, Peprotech,300‐07) were supplemented at 100 ng mL^−1^. Some constructs were immediately analyzed as day 0 controls and the rest were placed in 2 mL medium and cultured at 34 °C, 5% CO_2_, and 21% O_2_ for 7 days.

To confirm that hiPSC‐Sertoli cells have the capacity to mature and express tight junctions in vitro, fibers containing a high density of Sertoli cells were printed and cultured under conditions mimicking the pubertal environment. HiPSC‐Sertoli cells (iPS11‐10 line) were printed at 25 million mL^−1^ in either YIGSR or COL bioink with 0.05 mg mL^−1^ RA‐MS using a 22‐gauge nozzle. A portion of the cells were sacrificed to collect their ribonucleic acid (RNA) for quantitative reverse transcription polymerase chain reaction (RTqPCR) day 0 controls. The printed hiPSC‐Sertoli cells were cultured for 10 days at 34 °C and 5% CO_2_ in HPLM, 15% CTS KnockOut SR XenoFree supplement, 1 µM vitamin C, 1 µM vitamin E, 10 µg mL^−1^ biotin, 60 ng mL^−1^ progesterone, 60 µM putrescine, 30 µg mL^−1^ pyruvate, 30 ng mL^−1^ β‐estradiol, 1X MEM Vitamin Solution, 1X GlutaMAX Supplement, 1 µL mL^−1^ DL‐lactate, and 50 µM β‐mercaptoethanol, with various hormones and growth factors depending on the condition. Condition 1 consisted of 2 steps designed to promote growth followed by maturation. Constructs were cultured at 21% O_2_ with 20 ng mL^−1^ FSH, 10 ng mL^−1^ FGF2, 10 ng mL^−1^ Fibroblast Growth Factor 9 (FGF9), 20 ng mL^−1^ EGF, and 10 ng mL^−1^ Inhibin Subunit Beta A (Activin A) for 5 days, followed by 5 days at 10% O_2_ with 20 ng mL^−1^ FSH, 10 µM testosterone, 10 ng mL^−1^ T3, 50 ng mL^−1^ KITLG, 50 ng mL^−1^ BMP4, 10 ng mL^−1^ FGF2, and 20 ng mL^−1^ EGF. Condition 2 consisted of only the maturation step described above for 10 days.

### 2.7 Cell Tracker Live Cell Imaging and Core–Shell Measurements of Constructs

Live cell fluorescent tracking was done using CellTracker dyes (Thermofisher, C7025/C2925/C34565). Dyes were added to monolayer cell cultures in medium composed of HPLM (Gibco, A4899101), 1X GlutaMAX, and 1X ITS supplement at 5 µM for 45 min at 34 °C. The dye was then rinsed and replaced by regular medium. Cells were printed and imaged the same day. Each print was visually confirmed to have a core‐shell configuration prior to culture. Visualization and imaging were done with a FLUOVIEW FV3000 confocal microscope (Olympus). Scale bars were added in CellSens software (Olympus). Measurements were completed in ImageJ open‐source software using the Measurement Tool. Ten measurements from each image were taken from seven separate images for core and shell. Lengths of core and shell are represented as the averages and standard deviations.

### 2.8 Immunohistochemistry (IHC) of hiPSC‐Derived Somatic Cells

HiPSC‐derived cells were attached to slides coated in rhLaminin521 (Gibco, A29248) using 4‐well inserts (Ibidi, 80 466). Cells were seeded and cultured in HPLM with 5% human serum for 2 days to remove variability in expression caused by different culture conditions prior to analysis. Frozen testis tissue blocks in Tissue‐Tek Optimal Cutting Temperature (OCT) Compound (Sakura, 4583) were sectioned at 5 µm onto the slides alongside cultured hiPSC‐derived cells immediately prior to fixation of the slides for 15 min with 4% paraformaldehyde (PFA, Thermo Scientific, J19943‐K2). IHC was performed on the DISCOVERY Ultra Autostainer (Ventana Medical Systems). Antigen was retrieved with Cell Conditioning 1 (CC1, Ventana) at 95 °C for 64 min. Primary antibodies diluted in DISCOVERY Antibody diluent (Ventana Medical Systems, 760‐108) were applied for 16 min at room temperature to detect the following targets: SOX9, 1:1000 (Millipore Sigma, AB5535), DLK1, 1:4000 (Abcam, ab21682), and ACTA2, 1:200 000 (Invitrogen, 14‐9760‐82). Anti‐Rabbit UltraPolymer Horseradish Peroxidase (HRP, Cell IDx, 2RH‐050) or DISCOVERY UltraMap anti‐Rb HRP, Ventana, 760‐4315) and DISCOVERY ChromoMap DAB kit (Ventana Medical Systems, 760‐159) were used for detection. All tissues were counterstained in hematoxylin and bluing reagent. After washing in soapy water, tissue sections were dried and mounted with Cytoseal (Fisher Scientific, 22‐050‐262). For analysis, stained tissues were scanned with the Aperio AT2 scanner (Leica Microsystems) and viewed with ImageScope software (Leica Microsystems).

### 2.9 Immunocytochemistry (ICC) of hiPSC‐Derived SSCs

Cells were fixed for 10 min at room temperature in 4% PFA, then rinsed three times with 0.025% Tween20 (Sigma, 11 332 465 001) in PBS, then permeabilized for 10 min in 0.1% Triton X‐100 (Sigma, X100) in PBS at room temperature and rinsed three more times. The cells were then blocked for 1 h in 10% normal goat serum (NGS, Abcam, ab7481) in PBS. Primary antibodies were diluted in 10% NGS in PBS as follows: SSEA4, 1:100, (Abcam, ab16287), UTF1, 1:50 (Millipore Sigma, MAB4337), ID4, 1:50 (Invitrogen, pa5‐26976), SALL4, 1:500 (Abcam, ab29112), UCHL1, 1:500 (Abcam, ab108986), GFRA1, 1:500 (Abcam, ab18956), GPR125, 1:500 (Abcam, ab51705), DDX4, 1:250 (Invitrogen, PA5‐79147), NANOG, 1:100 (Sigma, N3038), DMRT1, 1:50 (Santa Cruz Biotechnology, sc‐377167), ZBTB16,1:100 (R&D Biosystems, MAB2944), and incubated overnight at 4 °C in the dark. Cells were rinsed 3 times the next day for 5 min each. Goat anti‐Rabbit Secondary Antibody Alexa Fluor Plus 488 (1:500, Thermofisher, A32731), Goat anti‐Mouse Secondary Antibody Alexa Fluor Plus 594 (1:200, Thermofisher, A32740), Goat anti‐Rabbit Secondary Antibody Alexa Fluor Plus 647 (1:200, Thermofisher, A32733) were diluted in 10% NGS in PBS and incubated 1 h at room temperature. Cells were rinsed another 3 times. 4′,6‐diamidino‐2‐phenylindole (DAPI, Abcam, ab228549) was diluted to 2.5 µM in PBS and added to the cells for 15 min in the dark at room temperature, and then replaced by PBS. Cells were imaged using a FV3000 confocal microscope, and scale bars added in CellSens software. To aid in visualization within the panel figures, ImageJ was used to brighten and increase the contrast. The same adjustment was applied equally to all images.

### 2.10 Whole Mount Immunofluorescent (IF) Staining of Constructs

IF and optical clearing were done as previously described, depending on the targets. Intracellular targets were stained according to the protocol described by Dekkers et al.^[^ ^14^ ^]^ In brief, constructs were fixed in 4% PFA for 45 min at 4 °C, rinsed for 10 min in PBS with 0.1% Tween20 and then blocked for at least 15 min in wash buffer, consisting of PBS with 0.1% bovine serum albumin (BSA, Miltenyi Biotec, 130‐091‐376), 1% Triton X‐100 (v/v), and 0.02% sodium dodecyl sulfate (SDS, Fisher BioReagents, BP1311‐1) at 4 °C. Constructs were incubated overnight at 4 °C on a rocking plate in primary antibodies, diluted in wash buffer as follows: TUBB3, 1:500 (Abcam, ab18207), Vimentin, 1:500 (Abcam, ab20346), COL4A1, 1:100 (R&D Systems, mab6308), LAMA1, 1:100 (Invitrogen, pa1‐16730). The next day they were rinsed with wash buffer for 2 h each at 4 °C on a rocking plate, then incubated overnight with secondary antibodies, DAPI and pre‐conjugated phalloidin, diluted as follows: Goat anti‐Rabbit Secondary Antibody Alexa Fluor Plus 488 (1:200), Goat anti‐Mouse Secondary Antibody Alexa Fluor Plus 594 (1:200), Goat anti‐Rabbit Secondary Antibody Alexa Fluor Plus 647 (1:200), AlexaFluor Plus 647 Phalloidin (1:200, Thermofisher, A30107), and DAPI (1:1000).

IF staining for surface targets was done using the protocol described by Beşikçioğlu et al.^[^ ^15^ ^]^ Briefly, constructs were fixed in 2% PFA for 15 min at room temperature, washed 4 times with 7.5 mg mL^−1^ glycine (Sigma Aldrich, 410 225) in PBS at 20 rpm, then 4 times in wash buffer consisting of 0.0048 µg mL^−1^ sodium azide, 0.0975 µg mL^−1^ BSA, 1.95 µL mL^−1^ Triton X‐100, and 0.4878 µL mL^−1^ Tween20 in PBS at 20 rpm. They were blocked for 1 h at room temperature in wash buffer with 2% NGS. Primary antibodies were diluted in wash buffer and incubated 48 h at 4 °C: KI67, 1:200 (Abcam, ab16667), ZO‐1, 1:200 (Invitrogen, 33‐9100), AR, 1:50, (Invitrogen, ma5‐13426), FSHR, 1:50 (Proteintech, 22665‐1‐ap), CX43, 1:100 (Invitrogen, 14‐4759‐80), PARD3, 1:200 (Millipore, 07‐330). Constructs were then washed 3 times with wash buffer at 20 rpm followed by overnight incubation at 4 °C with secondaries and DAPI as follows: Goat anti‐Mouse Secondary Antibody Alexa Fluor Plus 555 (1:500, Thermofisher, A32727), Goat anti‐Rabbit Secondary Antibody Alexa Fluor Plus 647 (1:500), DAPI (1:1000). Constructs were washed 4 times with wash buffer at 20 rpm and 4 times with PBS at 20 rpm.

Both protocols were completed by optical clearing using the FUnGI solution described by Dekkers et al. consisting of 50% (v/v) glycerol (Sigma Alrich, G9012), 9.4% (v/v) ddH_2_O, 10.6 mM Tris Buffer pH 8.0 (Sigma Aldrich, 648 314), 1.1 mM EDTA (Sigma Aldrich, 324 506), 2.5 M fructose (Sigma Aldrich, 0127), 2.5 M urea (Sigma Aldrich, U1250). Solution was removed as much as possible and replaced with fructose, urea and glycerol clearing solution for imaging (FUnGI) and allowed to sit at least 20 min at room temperature, then stored at 4 °C overnight before imaging.

Constructs were imaged using an FV3000 confocal microscope. Z‐stacks were collapsed into the maximum projection images and scale bars were added in CellSens software. ImageJ software was used to increase the brightness and contrast to improve visibility in the small panels.

### 2.11 Live–Dead Staining of Constructs

Live‐dead staining was done using a Cyto3D Live Dead Assay Kit (TheWell Bioscience, BM01) as per the manufacturer's instructions. This kit was chosen due to its ability to stain within full medium containing serum replacement rather than PBS, preserving cell viability during confocal acquisition of Z‐stacks. Briefly, 2 µL of stain was added per 100 µL of medium and incubated at 34 °C for at least 15 min. Images were taken using an FV3000 confocal microscope. Emission filters used were Green Fluorescent Protein (GFP) and Texas Red. Scale bars were added in CellSens software. To aid in visualization within the panel figures, ImageJ was used to brighten and increase the contrast. The same adjustment was applied equally to all images. Images are representative of each condition (n = 3).

### 2.12 Particle Templated Instant Partition (PIPseq) Single Cell RNA Sequencing

Cells were detached from cell culture plates by TrypLE Express and re‐suspended in Cell Suspension Buffer (Fluent Biosciences). Quantification and cell viability was determined by Trypan Blue solution (Sigma Aldrich, T8154). Cell concentration was adjusted to 1250 cell µL^−1^ using a Cell Suspension Buffer with 20U of Protector RNAse Inhibitor (Roche, 3 335 399 001). Using PIPseq IV T2 3′ Single Cell RNA Kit (Fluent Biosciences), 4 uL of cell suspension was input as per kit guidelines to approximate a final yield of 2000 cells per library.^[^ ^16^ ^]^ Cells were captured, and library preparation was carried out according to manufacturer's instructions (Fluent Biosciences). Libraries were converted to be compatible for sequencing using DNA Nanoball Sequencing (DNBSEQ)‐400 technology (Complete Genomics) with a DNBSEQ universal library conversion kit (Complete Genomics) according to manufacturer's instructions. Briefly, 50 ng of libraries were first amplified to add‐in adaptors and were denatured to obtain single‐stranded deoxyribonucleic acid (ssDNA) libraries, which were then circularized. Four libraries were pooled, and nanoballs were made from a total of 60 fmol ssDNA libraries using DNBSEQ High‐throughput Sequencing Primer Kit (App‐D, Complete Genomics) and loaded onto a DNBSEQ‐400 sequencing platform (Complete Genomics) to generate 400 million raw reads aiming for 50000 raw reads/cell.

### 2.13 Single Cell Sequencing Bioinformatics

To generate gene‐cell count matrices, raw FastQ files were aligned to the human genome (GRCh38.p13 (GENCODE v40 2022.04, Ensembl 106), and cell calling was performed using sensitivity level 3 in PIPseeker software (version 3.2.0). All downstream analyses were carried out using Seurat (version 5.1.0). Quality control was conducted to remove low‐quality cells. Cells were filtered based on the following criteria: minimum gene count per cell of 200, and minimum of 3 cells a gene needs to be detected in. The data was normalized and log‐transformed using the LogNormalize function in Seurat. Genes that had larger variance were chosen using Seurat FindVariableFeatures and subjected to Principal Component analysis (PCA) dimensionality reduction. The top 10 principal components were selected based on the elbow plot and variance contribution. Clustering was done by shared nearest neighbor (SNN) modularity with a resolution parameter of 0.3 to identify distinct cell populations. Clusters were visualized by uniform manifold approximation and projection (UMAP).

Cells were scored for cell cycle phases using CellCycleScoring function based on a list of cell cycle markers from Tirosh et al.^[^ ^17^ ^]^ Highly proliferative cells were distributed throughout clusters, showing clustering was not impacted by the influence of cell cycle on gene expression. Cell annotation was based on the expression of known marker genes and visualized in violin plots and feature plots. To identify the gene expression programs of the cells and cell subtypes, differentially expressed genes were determined using FindAllMarkers function with a fold change threshold of 2, and maximum cells per group down‐sampled to 200. The genes were considered as differentially expressed if they met the criteria of an adjusted *p*‐value < 0.01 and fold‐change >= 4. For gene set enrichment analysis the top 50 genes were used, based on p‐value after cutting at a fold change of 4 and ensuring that the corrected p‐values were <= 0.01.

The samples were also integrated with public datasets of primary testicular cells to map their similarity to specific ages (e.g., infant, prepubertal, adult).^[^ ^18^ ^]^ The count matrices were generated by mapping reads to the reference transcriptome (GRCh38‐2020‐A) using Cell Ranger (version 7.1.0) for public samples (adult, 14 years old, 13 years old, 11 years old, 7 years old, infant) and PIPseeker (version 3.3.0) for hiPSC samples.^[^ ^19^ ^]^ These matrices were subsequently loaded as Seurat objects and filtered based on the mitochondrial content and the number of genes per cell. Data normalization and scaling were performed using Seurat's NormalizeData and ScaleData functions, followed by PCA for dimensionality reduction using RunPCA function. Doublets were identified and removed using DoubletFinder (version 2.0.4).^[^ ^20^ ^]^ The nearest‐neighbor graph is constructed by the FindNeighbors function and used by FindClusters to generate the initial clusters. Clusters were labeled using sc‐type package (version 1.0) according to known marker genes (see Supporting Information).^[^ ^21^ ^]^ After Identifying cell types in public samples, specific cell types (e.g., Sertoli) were subsetted and integrated with the corresponding hiPSC sample. Finally, pseudotime analysis was performed by Slingshot (version 2.6.0), and the differentially expressed genes for each sample were calculated using the Seurat's FindAllMarkers function and generated heatmap using DoHeatmap function.^[^ ^22^ ^]^ In Leydig and Myoid cell types, the 7‐year‐old sample was excluded due to a low number of cells. In SSCs, the 11‐year‐old sample was excluded due to poor quality (see Supporting Information).

### 2.14 RTqPCR

RT‐qPCR validation was done using PrimePCR Assays (Bio‐Rad) with SYBR green chemistry. RNA was extracted using RNeasy Plus Micro Kit reagents (Qiagen,74034). Complementary Deoxyribonucleic Acid (cDNA) was generated using iScript Reverse Transcription Supermix (Bio‐Rad, 1708840) with a Tetrad2 Peltier Thermal Cycler (Bio‐Rad). PrimePCR Primers (Bio‐Rad) used were as listed in **Table** **1**. RT‐qPCR was done with SsoAdvanced Universal SYBR Green Supermix (Bio‐Rad, 1725270) on a LightCycler96 (Roche).

**Table 1:** Primer sequences used in RT‐qPCR.

Gene	Amplicon content sequence	Bio‐Rad unique assay ID
GAPDH	GTATGACAACGAATTTGGCTACAGCAACAGGGTGGTGGACCTCATGGCCCACATGGCCTCCAAGGAGTAAGACCCCTGGACCACCAGCCCCAGCAAGAGCACAAGAGGAAGAGAGAGACCCTCACTGCTGGGGAGTCCCTGCCACAC	qHsaCED0038674
AMH	TGGGGGCGCCGGGCACTGTCCCCCAAGGTCGCGGCAGAGGAGATAGGGGTCTGTCCTGCACAAACACCCCACCTTCCACTCGGCTCACTTAAG	qHsaCED0005192
DAZL	GAACATACTGAGTTATAGGATTCATCGTGGTTGTGGGCTGCATATAAGTTTCAGTGTTTGGATTAGTCCAGACATTCTGAAACTGTGGTGGAGGAGGATGATTAAAAACCAAAGGACGTGG	qHsaCED0042658
DMRTB1	CATCTTTCTCACTGACCGTCCTGTTTGATACTGACAAGGAGAACACTGATGACCA GGATGCAGAGGTACTGTCGGGTGAGCCCAGCCAGCCATCGTCT	qHsaCID0009804
DPPA3	GCTACGTTTCAAAGATCCTGGAGAAGCCTAGTGTTGTGTCAAGACGCCGATGGACCCATCACAGTTTAATCCAACCTACATCCCAGGGTCTCCACAAATGCTCACCGAAGAAAATTCCCGGGACGATTCAGGGG	qHsaCED0038968
FSHR	AAGTCATAGTCAAACTCAGTGTACGTCATGTCAAATCCTCTGCTGTAGCTGGACTCATTGTCTTCTGCCAGAGAGGATCTCTGACCCCTAGCCTGAGTCATATAATCAAC TTCTTGCCTTAAAATAGATTTGTTGCAAATTG	qHsaCED0046741
GDNF	AGAGCCGCTGCAGTACCTAAAAATCAGTTCCTCCTTGGTTTCATAGCCCAGACCC AAGTCAGTGACATTTAAATGTATTGCAGTTAAGACACAACCCCGGTTT	qHsaCED0046567
HSD17B3	GGAGAATCTCACGCACTTCGCCAGGCAGGCCAGGCACACCAGCAGCCCTGTGAGGATGAAGAACTGTTCCAGGACGTCCCCCATGGCTGCACTCAA	qHsaCED0044349
ID4	GTTTTGCCCAGTATAGACTCGGAAGTAACAGTTATAGCTAGTGGTCTTGCATGATTGCATGAGATGTTTAATCACAAATTAAACTTGTTCTGAGTCCATTCAAATGTGTTTTTTTAAATGTAGATT	qHsaCED0046882
KIT	GCGGCGCCTGGGATTTTCTCTGCGTTCTGCTCCTACTGCTTCGCGTCCAGACAG GCTCTTCTCAACCATCTGTGAGTCCAGGGGAACCGTCTC	qHsaCID0008692
KITLG	AATGTACGAAAGTAACAGTGTTGATACAAGCCACAATTACACTTCTTGAAACTCTCTCTCTTTCTCTTGCAACATACTTATCTCATTATCCTCTTCATTAATTTGTATATTTTC AACTGCCCTTGTAAGACTTGGCTGTCTCTTCT	qHsaCID0008103
LHCGR	CAGTTAACACTCTGTGTAGCGAGTCTTGTCTAGGAGAGCTGTACCTTGACAGTGCAATGTGGACAACTTCAAGGTGGATTGAGAAGGCTTATTTGATCCAGT	qHsaCED0044808
MLH3	CATGGCAGGCTTGGGATGCCAACACCTTCTGGACAGTCAGTGGCAATGTCCCTT GGATGCCTCCGGTGGTCTGGAGTAGCTCCAGTTGTTCTCGGATAAATTCCTCCA CAATACTCTTGGTCACAGTAGATCTTCCTCTCCGAAGTTCATTGGCTTC	qHsaCID0021156
NGF	GTGGTTCCGCCTGTATGCCGATCAGAAAAGCTGTGATCAGAGTGTAGAACAACATGGACATTACGCTATGCACCTCAGTGTGGCCAGGATAGAAAGCTGCTCCCTTGGTAAAAC	qHsaCID0016983
PRM2	GCCTCCTTCGAGAGCAGTGTCTGCGCCTATAGTGAGACTGGCCATGGGTCCTCT CGTAGACCTCGACGTGCTCCGGGCTCAGCCCTTGCTCCTCTTGGCCGTGGTGT CCTTGCTCTTGCCCATGCAACTGCTGCCTGTACACCTCGTGCGAGCGTTCGCTC AGGCTCCT	qHsaCED0002538
SOHLH2	TGGCTTCCAGCTCCTCGGCACTTAGTGAAGAAGGCACCTTCAAGAGAACCATGT TGAATATGCAATCATCCAAAAGCGCTGCTGCCTCCTTCGTGTCACTGATGGTGAT GGTGACTTCTGCTA	qHsaCED0037999
SOX9	AACCTTGGCTAAATGGAGCAGCGAAATCAACGAGAAACTGGACTTTTTAAACCCTCTTCAGAGCAAGCGTGGAGGATGATGGAGAATCGTGTGATCAGTGTGCTAAATCTCTCTGCCTGTTTGGACTTTGTAATTAT	qHsaCED0044083
STRA8	GCTCTTCAACAACCTCAGGAAGACAGTGTACTCTCAGTCTGATCTCATAGCCTCAAAGTGGCAGGTTCTGAATAAGGCAAAGAGTCATATTCCAGAACTGGAGCAAACCCTGGATAATTTGCTGAAGC	qHsaCID0016584
SYCP3	AGTCTTCCCTTCAATAACATCTTCCTCTGATCCACTCAGATCTTTCTTATCTTCAG TCTCAAAGTCATAGGCTCTCGTAAACTGATCTTCCACAGACGGCTTCCCA	qHsaCED0044910
TSPY4	GCTGCTGTTGGATGACATAATGGCGGAGGTGGAGGTGGTGGCGGAGGTGGAGGTGGTGGCGGAGGAGGAGGGCCTCGTGGAGCGGCGGGAGGAGGCCCAGCGGGCACAGCAGGCTGTGCCTGGCCCTGGGCCCATGACCCCAGAGTCTGCACTGGAGGAGCTGCTGGCCGTTCAGGTGGAGCTGGAGCCGGTTAATGCCCAAGCCAGGAAGGCCTTTTCTCGGCAGCG	qHsaCED0037465
UTF1	GCCGCCGCCGCTACAAGTTCCTTAAAGACAAGTTTCGCGAGGCGCACGGCCAGCCGCCCGGGCCCTTCGACGAGCAGATCCGGAAGCTCATG	qHsaCED0046843
ZPBP	CTTAAGTAAGGAAATTTCACATGAAAGGTCAAGAAGCAGTTTGCTTAAAATCTGA AGAAGTTTCTTCTCAAAAGAAATATTATAAATGCTATTGCAGGGAGCTGCATGATA TCGAGCTGTGAACTGATAATAATAATGAGGCTCACGATA	qHsaCED0042089

### 2.15 Statistics

Statistics were carried out in John's Macintosh Project (JMP) software (Statistical Analysis System (SAS) Institute), and graphs were generated in GraphPad software (Prism) A normal distribution was not assumed in the datasets, and therefore nonparametric tests were used. Specifically, medians of three technical replicates (separate constructs from each condition) were used to calculate statistical significance using the Wilcoxon Exact Test with α<0.05. GAPDH was used as a housekeeping gene to normalize qPCR data (calculate ΔCt). Each assay was run in technical triplicates. Grubb's Test was used to identify outliers. Expression was represented as the fold change between conditions (2^−ΔΔCt^).

## 3 Results

### 3.1 Strong Mesoderm Induction is Necessary for Testicular Lineage Differentiation from hiPSCs

**Figure 1:**
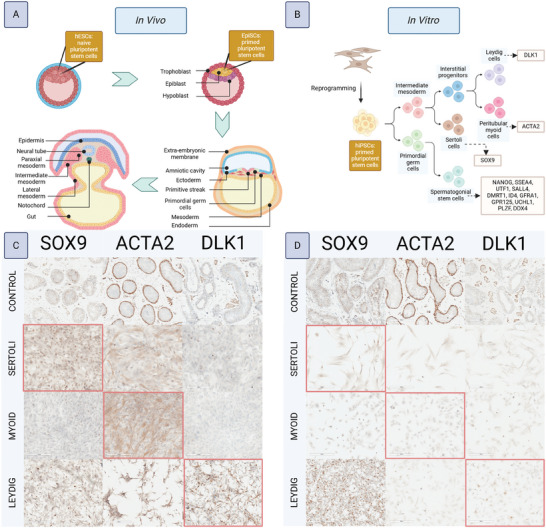
IH confirmation of somatic testis cell fate acquisition from hiPSCs. A) Schematic showing in vivo differentiation of testis cells from pluripotent stem cells, beginning with gastrulation of the blastocyst. Naive hESCs are present prior to gastrulation, whereas primed EpiSCs arise in the epiblast following gastrulation. The epiblast develops into ectoderm, endoderm, and mesoderm layers, as well as PGCs and an organizing center known as the notochord which secretes patterning signals. The primitive streak is an early precursor of the mesoderm layer which arises alongside PGCs. The mesoderm is patterned into lateral, intermediate, and paraxial areas. Somatic gonadal cells arise from the intermediate mesoderm layer, while PGCs migrate there. B) Schematic showing in vitro differentiation of testis cells from pluripotent stem cells. HiPSCs are reprogrammed from differentiated adult somatic cell types such as fibroblasts and possess characteristics of both naive and primed pluripotent stem cells. Their in vitro differentiation mimics that of in vivo. SSCs pass through a PGC state, while somatic testis cells pass through an intermediate mesoderm state which bifurcates into Sertoli cells and a single interstitial cell progenitor which gives rise to peritubular myoid and Leydig cells. C) 11‐10 hiPSC line acquisition of somatic fates, indicated by their adoption of phenotype markers: SOX9 for Sertoli cells, ACTA2 for peritubular myoid cells, and DLK1 for Leydig cells (indicated by the red boxes). The control is human adult testis sections freshly frozen in OCT, embedded, sectioned onto the same slides as the cultures, and then fixed and stained alongside them. D) Wicell hiPSC line acquisition of somatic fates. N = 1.

The prepubertal testis niche is populated by immature SSCs and immature somatic cells including Sertoli, Leydig, and peritubular myoid cells.^[^ ^23^ ^]^ Vasculature cells such as endothelial and macrophage cells are present but proven expendable in rodent IVS and so were excluded from this model for simplicity.^[^ ^24^ ^]^ An important consideration in hiPSC differentiation is that individual lines inherit variable lineage biases as a consequence of genetic differences and memory of their previous identities.^[^ ^25^ ^]^ It was hypothesized that since human testis cells arise from the early mesoderm, an hiPSC line biased toward mesoderm fates would provide the best starting material for testis differentiation (**Figure** **1**A).^[^ ^26^ ^]^ To that end, a male hiPSC line with a mesoderm bias was chosen and compared to a male hiPSC line with an ectoderm bias.^[^ ^13a^ ^]^

HiPSC derivation into Sertoli, peritubular myoid, and Leydig cell cultures was done using previously established protocols recapitulating in vivo patterning.^[^ ^13a,b^ ^]^ In short, the stem cells pass through an intermediate mesoderm phase into early gonadal progenitors which then bifurcate into pre‐Sertoli cells or interstitial progenitors (Figure 1B). Efficiency was assessed by immunostaining for expression of phenotype markers. SOX9 marks Sertoli cells, DLK1 marks Leydig cells, and ACTA2 marks peritubular myoid cells.^[^ ^19^, ^27^ ^]^ Each was stained for all markers alongside sections from adult testicular tissue as a positive control. In the mesoderm‐biased line, SOX9 expression was predominantly expressed in hiPSC‐Sertoli cells, DLK1 in hiPSC‐Leydig cells, and ACTA2 in hiPSC‐peritubular myoid cells (Figure 1C). In the ectoderm‐biased line, a similar pattern of expression was observed but with substantially lower efficiency suggesting incomplete differentiation (Figure 1D).

**Figure 2:**
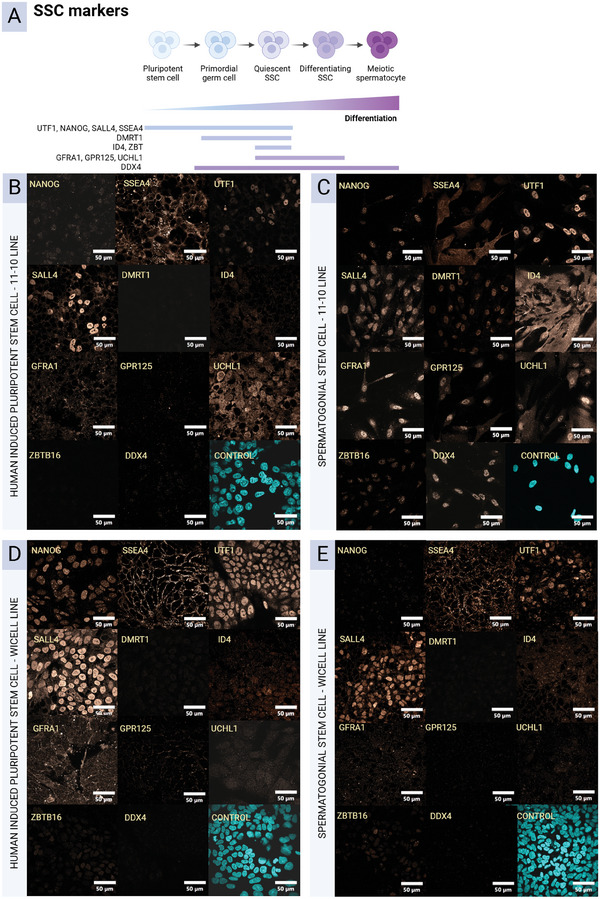
ICC confirmation of SSC differentiation from hiPSCs. A) Phenotype markers and their expression amongst the states of germ cell differentiation. B) 11‐10 hiPSC line undifferentiated control. C) 11‐10 hiPSC line acquisition of SSC markers following differentiation. D) Wicell hiPSC line undifferentiated control. D) Wicell hiPSC line acquisition of SSC markers following differentiation. N = 1.

SSCs were generated using a specialized medium formulation discovered to differentiate hiPSCs directly into SSCs.^[^ ^28^ ^]^ SSCs can adopt multiple phenotypes; therefore, a larger set of markers was needed to assess SSC fate acquisition (**Figure** **2**A). HiPSCs and SSCs share markers related to pluripotency so undifferentiated hiPSCs served as a control. Pluripotent markers NANOG, SSEA4, UTF1 and SALL4 are expressed in both hiPSCs and SSCs; while DMRT1 marks primordial germ cells (PGCs) and SSCs; ID4, GFRA1, GPR125, UCHL1, and ZBTB16 (PLZF) mark SSCs; and DDX4 marks all germ cells.^[^ ^9^, ^19^, ^23^, ^29^ ^]^ The mesoderm‐biased hiPSCs shifted from UTF1^+^ / NANOG^+^ / SALL4^+^ / SSEA4^+^ / UCHL1^+^ expression to UTF1^+^ / NANOG^+^ / SALL4^+^ / SSEA4^+^ / DDX4^+^ / ID4^+^ / ZBTB16^+^ / GFRA1^+^ / GPR125^+^ / DMRT1^+^ / UCHL1^+^ expression, indicating a transition toward an SSC‐like fate (Figure 2B,C). The ectoderm‐biased hiPSCs shifted from UTF1^+^ / NANOG^+^ / SALL4^+^ / SSEA4^+^ / GFRA1^+^ expression to UTF1^+^ / SALL4^+^ / SSEA4^+^ / ZBTB16^+^ expression, indicating a partial induction closer to hiPSC than SSC (Figure 2D,E).

Overall, acquisition of testicular lineages was efficient in the mesoderm‐biased hiPSC line (11‐10), whereas it was not in the ectoderm‐biased hiPSC line (Wicell), supporting the hypothesis that strong mesoderm induction is necessary for efficient testis fate acquisition.

### 3.2 Single cell sequencing reveals subpopulations present within hiPSC‐derived testicular cultures

**Figure 3:**
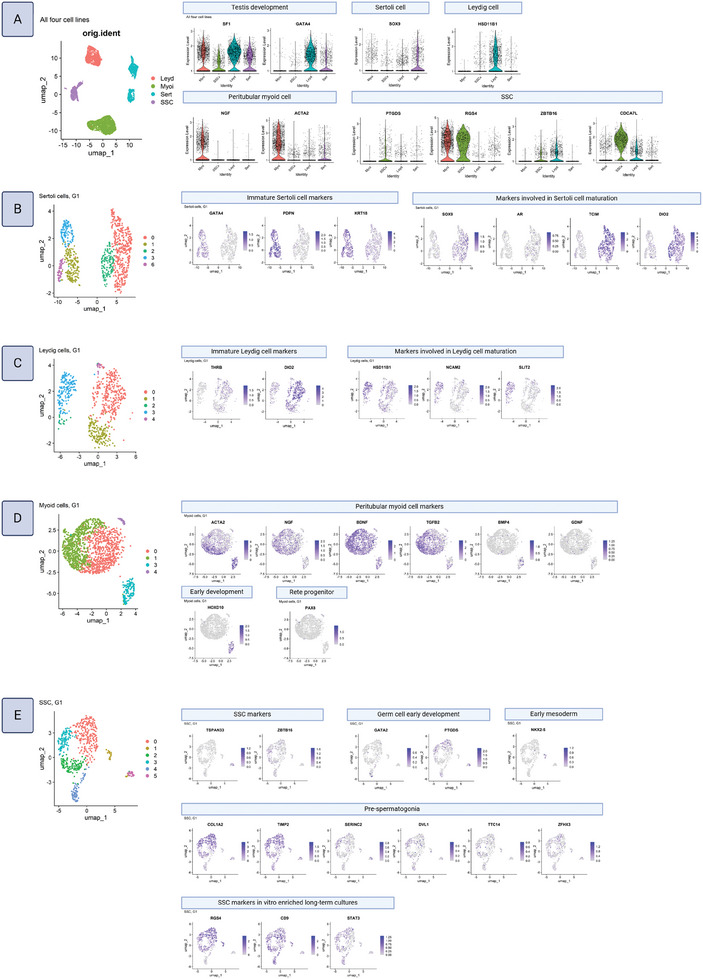
Single cell RNA sequencing reveals subpopulations present within each culture. UMAPs and feature plots of key genes for A) all 4 datasets combined (Sertoli, Leydig, peritubular myoid, and SSC), B) hiPSC‐Sertoli cells, C) hiPSC‐Leydig cells, D) hiPSC‐myoid cells, and E) hiPSC‐SSCs.

Recently, single‐cell RNA sequencing has identified the presence of somatic and germ‐cell subpopulations with distinct transcriptomic profiles within the human testis.^[^ ^30^ ^]^ Therefore, to investigate the presence of subpopulations in the hiPSC‐derived testicular cultures, single‐cell RNA sequencing was performed from ≈2000 cells from each culture. Single cell data was projected onto a UMAP, revealing four distinct cell clusters (**Figure** **3**A). Each cluster was annotated based on the expression of well‐known markers corresponding to the expected cell types (SSC, Sertoli cells, Leydig cells, peritubular myoid cells). *SF1* and *GATA4* were used to annotate cells of testicular somatic fates, *SOX9* Sertoli cells, *HSD11B1* Leydig cells, and *NGF* and *ACTA2* peritubular myoid cells.^[^ ^31^ ^]^ Annotating the SSCs using well‐known markers was less straightforward. Canonical SSC markers including those observed in ICC were notably absent with the exception of the self‐renewal regulators *ZBTB16* and *CDCA7L*.^[^ ^32^ ^]^ However, the early differentiation gene *PTGDS* was highly expressed in the SSC cluster. In mouse development *PTGDS* acts to promote mitotic arrest in differentiating germ cells while downregulating pluripotency genes and upregulating germ cell genes.^[^ ^33^ ^]^ One study reporting on the transcriptional profile of human SSC cultures during long‐term in vitro expansion conditions (>3 months) showed that the transcript levels of many canonical SSC markers become low over time, coincident with the upregulation of markers associated with extracellular matrix (ECM) organization.^[^ ^34^ ^]^ One such marker from that study is *RGS4*, which was highly expressed in the hiPSC‐SSC cluster as well as the peritubular myoid cell cluster. *RGS4* is a G protein signal regulator most associated with connective tissue cells. Taken together, the annotation markers describe an hiPSC‐SSC transcriptional state dominated by ECM organization, self‐renewal, and early germ cell differentiation. The relatively low expression of markers for the maintenance of stemness (*UTF1*, *SSEA4*, *SALL4*, *NANOG*) and SSC state (*DMRT1*, *GFRA1*, *GPR125*, *DDX4*, *UCHL1*) may be explained by a lack of regulatory signaling from somatic cell types and other in vivo cues that can perturb their state. Likewise, the in vitro environment lacks the ECM complexity of the in vivo niche, and this may explain activities surrounding adhesion and ECM generation.

To analyze the subpopulations within each cell type, single‐cell RNA sequencing data was examined for each cell type separately. To determine whether the observed clustering was influenced by the cell cycle variation, cells were first scored based on the expression of cell cycle genes. Each cell subtype contained cells with high cell cycle scores, indicating their proliferative state, thus cell cycle effects did not account for the primary clustering patterns. This supported the biological relevance of the identified subtypes. To gain a better understanding of the transcriptional programs underlying the heterogeneity, we compared the differentially expressed genes (DEGs). Gene set enrichment analysis (GSEA) identifies coordinated changes in predefined gene sets, allowing the discovery of functional pathways and processes unique to cell types and subtypes. Therefore, to gain a better understanding of the biological significance of DEGs of cell subtypes at the pathway level, we performed GSEA of gene ontology terms on the DEGs of each cell subtype (see Supporting Information for cell cycle plots, DEG lists, and GSEA barplots).

Sertoli cells subclustered into 2 main subpopulations (Figure 3B). The top pathways for the first subpopulation described ECM organization and tubulogenesis, while the top DEGs included the immature markers *GATA4*, *PDPN*, and *KRT18*. Top pathways for the second subpopulation described ECM structure, cell junction assembly, and negative regulation of canonical Wnt signaling. Surprisingly, pathways for ossification and bone mineralization were also present. This may reflect an aberrant rare Sertoli cell behavior typically affecting prepubertal boys in which aromatase is overexpressed, resulting in higher than normal levels of estrogen, a hormone that also regulates bone growth.^[^ ^35^ ^]^ Estrogen also promotes prepubertal Sertoli cell proliferation and is supplemented in vitro.^[^ ^36^ ^]^ This finding may reflect that levels of estrogen supplementation in Sertoli cell cultures need to be carefully optimized in the future. Top DEGs included *AR*, the receptor for testosterone, and *DIO2*, an enzyme responsible for the local production of T3 hormone, both of which trigger maturation during puberty, and *TCIM*, a regulator of canonical Wnt signaling which is reported to inhibit Sertoli cell maturation.^[^ ^36^, ^37^ ^]^ Based on these observations the first subpopulation was characterized as immature, and the second subpopulation as an intermediate state showing signs of early maturation.

Leydig cells similarly subclustered into 2 main subpopulations (Figure 3C). Top pathways for the first subpopulation included cell‐matrix adhesion and fibroblast migration. Cold‐induced thermogenesis pathways were also listed, suggestive of carnitine metabolic activity since this pathway is most associated with brown fat cells and their use of carnitine to fuel heat production. Indeed, carnitine is reported to act as an antioxidant in protecting the testosterone production pathway in Leydig cells and they are reported to possess large stores of its derivative acylcarnitine.^[^ ^38^ ^]^ Top DEGs included the thyroid hormone enzyme *DIO2* and its receptor *THRB*. Thyroid hormones are active regulators of Leydig cells, triggering their differentiation from mesenchymal progenitors, promoting proliferation, and directly activating *STAR*, a major regulator of steroidogenesis.^[^ ^39^ ^]^ In the second subpopulation, top DEGs included *HSD11B1*, an enzyme that controls levels of glucocorticoid to support testosterone production and is a known marker of intermediate and adult‐state Leydig cells.^[^ ^31^, ^40^ ^]^ *SLIT2* was also in the top DEGs and is a negative regulator of testosterone production, in part through suppressing *LHCGR* expression, the receptor responsible for activating *STAR*.^[^ ^41^ ^]^ Top pathways included cell adhesion and immune cell migration. The latter is explained by top DEGs such as *SLIT2*, *CDH13*, *NCAM2*, and *ICAM*, which are known for their functions as both immune cell adhesion sites and neuronal axon guidance.^[^ ^42^ ^]^ Leydig cells are transcriptionally similar to neuronal cells and observed to develop numerous interdigitations between resident macrophage cells which help to support steroidogenesis, and so these pathways may point toward their actin organization and functional cell‐cell coupling.^[^ ^43^ ^]^ Overall, the first subpopulation was classified as immature, whereas the second subpopulation appeared to be maturing.

Peritubular myoid cells presented three subclusters: one major cluster with two much smaller subclusters (Figure 3D). The main subcluster expressed the smooth muscle gene *ACTA2* and the paracrine factors *NGF*, *BDNF*, and *TGFB2*, whereas the two small subclusters expressed higher levels of the transcription factors *BMP4* and *HOXD10*, or *PAX8*, respectively, indicating earlier states of development. *PAX8* is a known rete testis progenitor marker, indicating that the smallest subpopulation may represent an incorrect fate adoption.^[^ ^44^ ^]^ Top pathways for the *ACTA*/*NGF*/*BDNF*/*TGFB2* subcluster included regulation of cell growth, muscle development, exocrine system development, and *NGF* signaling. Top pathways for the *BMP4*/*HOXD10* subcluster included skeletal system morphogenesis and urogenital system development, while the *PAX8* subcluster was too small to determine top pathways. The main subpopulation was characterized as immature, and indeed the mature smooth muscle marker *MYH11* was not observed, whereas the small subpopulations appeared to be progenitors.^[^ ^44^, ^45^ ^]^

SSCs subclustered into 1 main subpopulation with 3 much smaller subpopulations (Figure 3E). The main subpopulation expressed the SSC self‐renewal markers *ZBTB16* and several known pre‐spermatogonia markers including *PTGDS*, *COL1A2*, *TIMP2*, *SERINC3*, *DVL1*, *TTC14*, and *ZFHX4*.^[^ ^29^, ^33^, ^46^ ^]^ It also showed strong expression of *CD9* and *STAT3*, which are regulators of cell growth and adhesion found to be upregulated in long‐term SSC cultures.^[^ ^34^ ^]^ Top pathways included canonical Wnt signaling, response to *TGFB* stimulus, cell junction assembly, and stem cell fate commitment. Canonical Wnt signaling and *TGFB* are mediators of SSC proliferation.^[^ ^46^, ^47^ ^]^ The smaller subpopulations appeared to represent an early mesoderm state (*NKX2.5*), a PGC state (*GATA2*) and an SSC state (*TSPAN33*).^[^ ^19^, ^23^, ^48^ ^]^ The top pathways for the second largest cluster, the *GATA2* subpopulation, were embryonic organ development and regionalization, mesenchyme development, and regulation of endothelial cell differentiation, fitting with a state closer to PGC than pre‐spermatogonia. Intriguingly, the top pathways for the *TSPAN33* subpopulation were muscle cell and myotube developmental pathways, and indeed, *MYOD* and other genes associated with sarcomeres and striated muscle were strongly represented in the top DEGs. Further examination showed that the subpopulation did not express smooth muscle markers such as *TAGLN*, *ACTA2*, and *MYL9*, confirming a skeletal muscle‐like profile. Although *TSPAN33* is a well‐known SSC marker in vivo, skeletal muscle expression amongst that subpopulation has never been reported; therefore, this muscle‐like transcriptional property appears to be unique to in vitro SSCs. Overall, the majority of the SSCs clustered into a pre‐spermatogonia transcriptional profile characterized largely by proliferation and immature ECM deposition.

**Figure 4:**
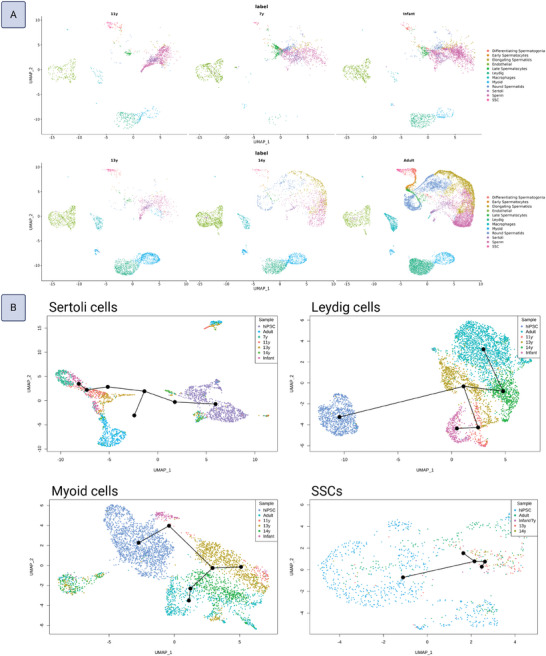
Integration with human testis tissue datasets and pseudotime trajectories. A) Cell type labeling of the public human testis single‐cell sequencing datasets from samples of various ages of development (infant, 7 years old, 11 years old, 13 years old, 14 years old, and adult). B) Pseudotime trajectory plots of each cell type integrated with the corresponding hiPSC‐derived sample.

The sample datasets were integrated with public datasets of human testis tissues (adult, 14 years old, 13 years old, 11 years old, 7 years old, infant) in order to further examine their developmental state using pseudotime trajectory analysis (**Figure** **4**A). In Leydig and peritubular myoid cell types, the 7‐year‐old sample was excluded due to a low number of cells; and in SSCs, the 11‐year‐old sample was excluded due to poor quality (high stress‐related gene expression, see Supporting Information). Following cell type annotation of the public datasets using their known published markers, each cell type was subsetted and combined with the corresponding hiPSC‐derived cell type (Sertoli, Leydig, peritubular myoid, SSC). The developmental trajectory of each integrated cell type was then visualized using pseudotime analysis (Figure 4B). HiPSC‐derived Sertoli cells mapped just before the 14‐year old sample, indicating a peripubertal state in keeping with the prior observation that they possess both immature and maturing subpopulations. Similarly, hiPSC‐derived Leydig cells, which were also observed to split into immature and maturing subpopulations, mapped just before the 13‐year old sample. HiPSC‐derived peritubular myoid cells mapped prior to infant samples, indicating a state closer to neonatal, in line with the prior observation of the main subpopulations being composed of immature smooth muscle cells and progenitors. Intriguingly, hiPSC‐SSCs mapped just prior to the 14‐year old sample, illustrating that, in comparison to in vivo SSCs, they were closer to a peripubertal developmental state than to pre‐spermatogonia.

### 3.3 Akt Inhibition Preserves the Quiescent State of hiPSC‐Derived SSCs

**Figure 5:**
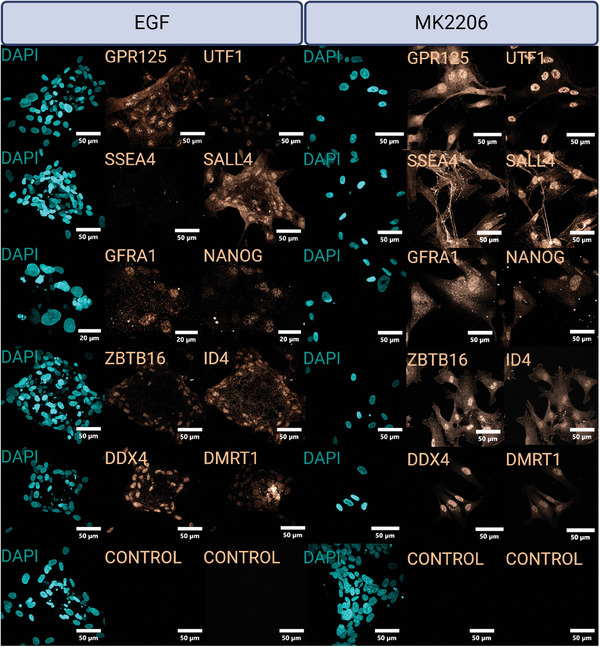
Immunostaining for SSC markers following the replacement of the neonatal mouse SSC growth factor EGF with Akt‐inhibitor MK2206 in hiPSC‐SSC cultures.

A prerequisite to realizing the hiPSC‐derived model was the determination of culture conditions to support SSC expansion. Conditions for mouse neonatal SSC culture have proven not to translate to human SSC culture.^[^ ^34^, ^49^ ^]^ This includes the growth factors FGF2, GDNF, EGF, and LIF, although LIF is observed to be unnecessary.^[^ ^50^ ^]^ In mice, SSC self‐renewal is dependent on Akt pathway activation, whereas in humans, recent findings suggest that Akt pathway activation favors SSC differentiation.^[^ ^51^ ^]^ Furthermore, the Akt pathway inhibitor MK2206 was observed to improve human SSC quiescence in vitro in the presence of FGF2 and GDNF. Therefore, we cultured hiPSC‐SSCs for 14 days with GDNF, FGF2, and either MK2206 or EGF to determine the better condition for hiPSC‐SSC quiescence and growth in vitro (**Figure** **5**). Replacing MK2206 with EGF accelerated hiPSC‐SSC growth but increased clustering behavior. Immunostaining revealed a loss of quiescent marker expression in the clusters alongside the appearance of apoptotic nuclei. In the cluster peripheries, quiescent markers NANOG, UTF1, ZBTB16, ID4, GPR125, GFRA1, and DDX4 were weaker with EGF, while SSEA4 was entirely lost. These findings suggest that the MK2206 condition was best for maintaining the quiescent SSC phenotype in hiPSC‐SSC culture.

**Figure 6:**
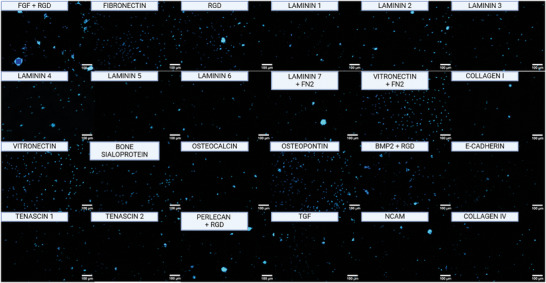
Matrix screen of 24 different biomimetic xeno‐free matrices. HiPSC‐SSCs stained for DAPI to visualize growth and spheroid formation.

HiPSC‐SSCs were unable to attach to tissue culture‐treated plasticware but could attach to laminin‐coated plasticware.^[^ ^49^ ^]^ However, culture on rhLaminin521 resulted in spheroid formation and eventual release from the plate (data not shown). Therefore, we screened a set of chemically defined biomatrices for an alternative ECM to support hiPSC‐derived SSCs. The biomatrices were composed of dextran‐conjugated starPEG hydrogels functionalized with ECM peptide motifs (screenMATRIX; denovoMATRIX, GmbH). The screen contained 24 combinations of peptides, (**Figure** **6**). After 4 days, hiPSC‐SSCs formed prominent clusters in response to peptide combinations FGF‐RGD, perlecan‐RGD, and laminin 7‐FN2, whereas monolayer growth was maintained on fibronectin, vitronectin, and the combination of vitronectin‐FN2. Of those options, vitronectin‐FN2 was chosen for expansion for the remainder of the study since it prevented clustering and since FN2 contains an FGF receptor binding motif and FGF signaling is reported to improve human SSC survival in vitro.^[^ ^51c^ ^]^

## 4 3‐D Bicompartmental Model

Coaxial bioprinting was used to generate the core‐shell geometry of the 3‐D bicompartmental model. HiPSC‐Sertoli cells and hiPSC‐SSCs were mixed into the core bioink to represent the tubular compartment of the testis niche, while hiPSC‐Leydig and hiPSC‐peritubular myoid cells were mixed into the shell bioink to represent the interstitial compartment. To isolate the effects of the core‐shell cytoarchitecture, core‐only constructs were printed as controls. As an additional negative control, the incompletely differentiated testis cells (from Wicell hiPSC line) were printed as well. The Sertoli cell to SSC ratio was chosen to reflect the ratio in vivo which ranges from an average of 4.1 Sertoli cells per SSC in puberty to 1.9 in adults,^[^ ^52^ ^]^ while the Sertoli cell to Leydig cell ratio was chosen arbitrarily since in vivo ratios vary widely, and the peritubular myoid to Sertoli cell ratio was chosen to reflect in vivo ratio of ≈1:1.^[^ ^53^ ^]^ The final concentrations were 3 million mL^−1^ SSCs, 4 million mL^−1^ Sertoli cells, 8 million mL^−1^ peritubular myoid cells, and 16 million mL^−1^ Leydig cells. To improve in vivo‐like cellular activity in the constructs, the β‐laminin adhesion motif YIGSR was incorporated into the core bioink, and the collagen adhesion motif COL was incorporated into the shell bioink. These motifs were chosen because β‐laminin is reported to stimulate SSC homing and Sertoli cell cord formation, while collagen is reported to stimulate Leydig cell steroidogenesis.^[^ ^54^ ^]^

### 4.1 Core–Shell Constructs Mimic Testis‐Like Dimensions and Mechanical Properties

**Figure 7:**
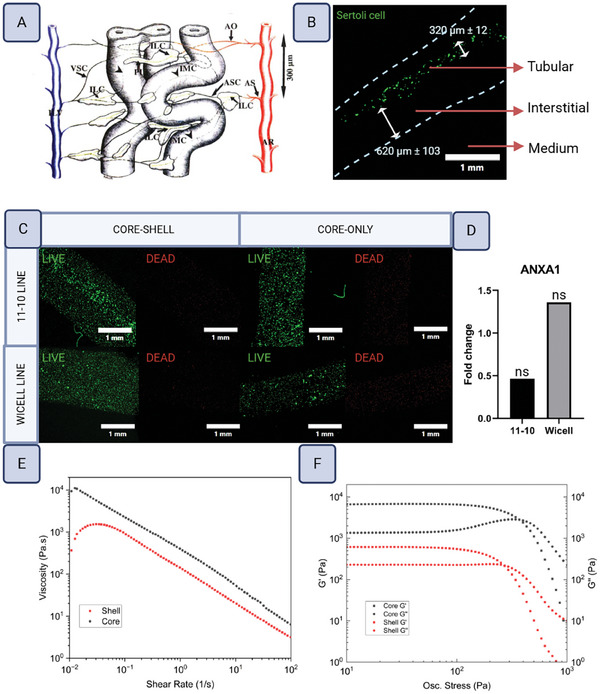
Core‐shell constructs. A) Computer‐aided 3‐D reconstruction of the human testis based on semi‐thin sections, showing microvasculature in correlation to Leydig cells and seminiferous tubules. AS = segmented artery, AR = recurrent artery, AO = arteriole, LC = Leydig cells, PLC = peritubular Leydig cells, ILC = inter‐Leydig capillaries, ASC = arterial side capillaries, IMC = intramural capillaries, VSC = venous side capillaries, ILV = intralobular veins. Reproduced with permission from John Wiley and Sons from *Microvasculature of the human testis in correlation to Leydig cells and seminiferous tubules”* by S. Ergun, J. Stingl, and A.F. Holstein. B) Dimensions of the core‐shell constructs visualized by confocal microscopy using fluorescent‐labeled hiPSC‐Sertoli cells. Dotted outlines of the constructs were traced from the phase contrast channel of the images. Dimensions are the means with standard deviations. N = 70 core‐shell constructs, n = 20 core‐only constructs. C) Live/dead staining of the constructs following 7 days culture. N = 3. D) *ANXA1* expression, presented as the fold change of core‐shell constructs compared to the core‐only condition after 7 days. The Wicell line was incompletely differentiated, acting as an additional control. N = 3. Statistical significance calculated by Wilcoxon Exact test, α = 0.05, * = significant, ns = not significant). E) Continuous ramp viscosity tests were carried out on the core (YIGSR, black) and shell (COL, red) bioinks at the shear rate from 0.01 to 100 s^−1^. F) Stress and strain sweep tests were carried out for the core (YIGSR, black) and shell (COL, red) bioinks to generate graphs of G’ and G’’.

In vivo, seminiferous cord diameters increase from 50–140 to 75–275 µm during puberty, while the distance between seminiferous tubules and major blood vessels has been measured at ≈500 µm (**Figure** **7**A).^[^ ^53^, ^55^ ^]^ Based on these dimensions, it was reasoned that a core diameter of ≈275 µm and a shell thickness of ≈500 µm would be ideal dimensions to mimic tissue‐like cell cross‐talk between core and shell compartments within the bicompartmental model. To visualize the core and shell dimensions, Sertoli cells were labeled using a membrane‐permeant fluorescent dye, and the constructs were imaged using confocal microscopy. Core diameters of the constructs measured 320 ± 12 µm, with shell thickness of 620 ± 103 µm (Figure 7B).

Following 7 days of culture, viability was assessed by live/dead staining. All constructs exhibited strong viability (Figure 7C). To ascertain whether the core‐shell cytoarchitecture had any effect on apoptosis, transcript levels for the apoptotic marker Annexin A1 (*ANXA1)* were measured and compared with core‐only constructs. *ANXA1* expression was decreased in the core‐shell constructs, indicating that the core‐shell architecture had a positive effect on cell viability. Conversely, *ANXA1* expression increased in the constructs of the incompletely differentiated control condition. Neither of the *ANXA1* changes were statistically significant (Figure 7D).

The rheological properties of the bioinks used for core and shell were characterized to better understand their printability and compare their softness to in vivo testis. As shown from the viscosity versus shear rate test, both core and shell bioinks showed shear thinning properties of greatly reduced viscosity at higher shear rate, which is essential for extrusion during bioprinting (Figure 7E). In addition, lower viscosity was observed for the shell ink as compared to the core ink, indicating the shell was softer than the core. To further understand the mechanical properties of the bioinks, the dynamic modulus was characterized using an oscillation stress/strain sweep test (Figure 7F). Storage modulus (G’) is a measure of the energy required to distort a sample, while loss modulus (G’’) is a measure of the energy loss and describes the viscous portion of the sample. In general, a higher storage modulus compared to the loss modulus indicates a gel‐like structure, which is essential for 3D printing. According to the stress and strain sweep tests, the shell bioink was again confirmed to be softer than the core bioink. At low stress, the core bioink had a G’ of 6000 Pa and a G’’ of 1500 Pa, while the shell bioink had a G’ of 600 Pa and a G’’ of 200 Pa. Comparatively, both bioinks exhibited similar softness to native testis tissue, which has a G’ ≈3000 Pa and G’’ and ≈1000 Pa.^[^ ^56^ ^]^ The point at which G’ meets G’’ shows the pressure where the bioink changes from solid to viscous liquid, and this transition facilitates extrusion bioprinting and indicates pressure levels acting on the cells, which if too high can induce toxicity. For both bioinks, this transition occurred at ≈300 Pa.

### 4.2 Core–Shell Constructs Promote Cytoskeletal Activity and Deposition of Collagen IV and α‐laminin

**Figure 8:**
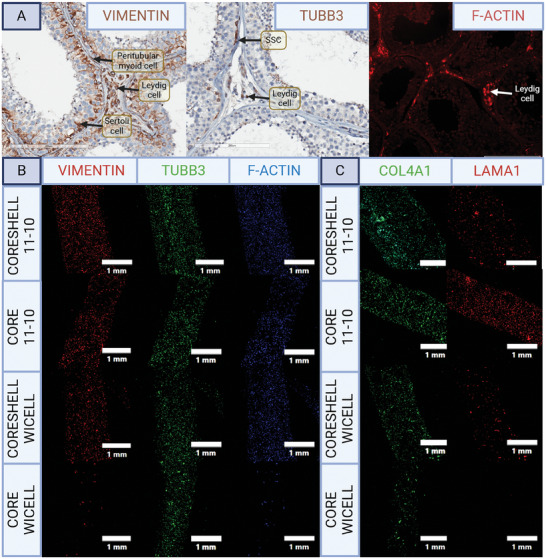
Cell‐matrix interactions. A) Immunostaining for cytoskeletal filaments in adult human testis. B) Immunostaining for cytoskeletal filaments in the constructs. C) Immunostaining for ECM deposition in the constructs, N = 3.

Tissue cytoarchitecture is secondarily defined by ECM, which harbors bioactive domains that bind to cellular receptors to activate cytoskeletal expression, regulating activities critical to tissue morphogenesis such as migration, division, polarization, and ECM remodeling.^[^ ^57^ ^]^ The normal expression of cytoskeletal elements in vivo was confirmed by immunostaining human adult testis which showed F‐actin expression in Leydig cells, β‐tubulin (TUBB3) expression in SSCs and Leydig cells, and vimentin expression in Sertoli, Leydig, and peritubular myoid cells (**Figure** **8**A). To confirm cytoskeletal expression in the core‐shell constructs, immunostaining was performed for F‐actin, β‐tubulin, and vimentin. All were expressed strongly in both the cores and shells of the constructs, with β‐tubulin more prevalent in the cores (Figure 8B). Expression was comparably sparse in the incompletely differentiated controls.

Collagen IV and α‐laminin are the major ECM molecules in the prepubertal testis cords so their deposition by the hiPSC‐derived cells within the constructs was assessed.^[^ ^58^ ^]^ Both collagen IV and α‐laminin were observed in the cores and shells of the constructs by immunostaining (Figure 8C). The incompletely differentiated controls deposited collagen IV and α‐laminin in the shells to a lesser degree, and failed to express α‐laminin in the cores.

### 4.3 Core–Shell Construct Cytoarchitecture Promotes Tubular‐Interstitial Crosstalk Reflective of Immature Morphogenic Activity

**Figure 9:**
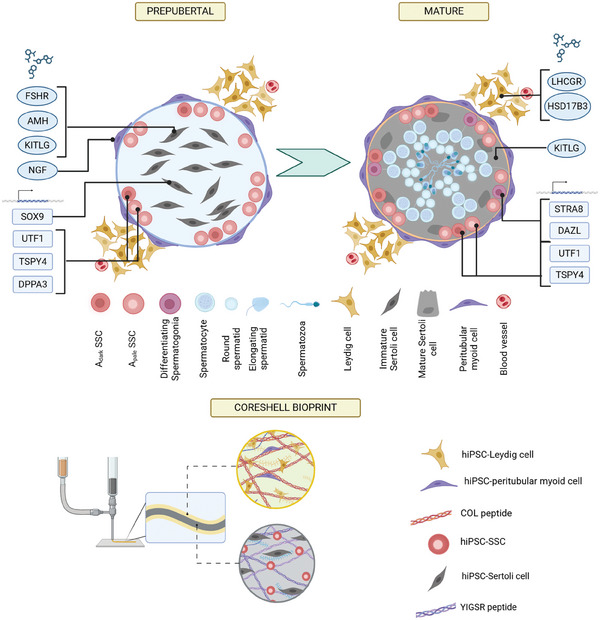
Schematic showing the cellular composition of prepubertal and mature testis and their expression of key genes regulating signaling factors and transcription factors (top). Schematic showing the creation and cellular composition of the hiPSC‐derived core‐shell model using coaxial bioprinting to mimic the bicompartmental makeup of prepubertal testis tissue with synthetic peptides COL and YIGSR to mimic collagen and laminin extracellular matrix (bottom).

Cellular cross‐talk between the core and shell compartments was characterized by examining changes in transcription between core‐shell constructs and core‐only constructs (**Figure** **9**). To observe shell compartment activity, interstitial cell markers *LHCGR*, *HSD17B3*, and *NGF* were examined. *LHCGR* encodes a surface receptor on Leydig cells that binds the endocrine signal LH, stimulating their maturation and steroidogenesis, while its loss results in delayed or absent puberty. *HSD17B3* encodes an enzyme necessary for testosterone synthesis expressed upon Leydig cell maturation.^[^ ^31^, ^59^ ^]^ Thus, *LHCGR* and *HSD17B3* transcription are indicators of mature interstitial steroidogenic activity. Conversely, *NGF* encodes a growth factor involved in testis cord morphogenesis, expressed by Leydig and peritubular myoid cell progenitors as well as fetal Sertoli cells, and therefore indicates immature interstitial compartment activity.^[^ ^45^, ^60^ ^]^ To investigate core compartment character, Sertoli cell genes *SOX9*, *AMH*, *FSHR*, and *KITLG* were targeted. *SOX9* encodes a transcription factor initiated in Sertoli cell progenitors which governs testis morphogenesis until puberty.^[^ ^23^, ^61^ ^]^ One of its direct targets is *AMH*, which encodes a secreted hormone that suppresses the development of female reproductive organs.^[^ ^62^ ^]^ Together, the activity of *SOX9* and *AMH* are characteristic of immature Sertoli cells.^[^ ^27a^ ^]^ Likewise, *FSHR* encodes an important immature Sertoli cell receptor for the endocrine signal FSH, whose action is responsible for stimulating Sertoli cell proliferation during cord morphogenesis.^[^ ^52^, ^63^ ^]^ Meanwhile, *KITLG* encodes a growth factor produced by Sertoli cells critical for PGC proliferation in the fetal stage and spermatogenesis post‐puberty.^[^ ^29^, ^64^ ^]^ It's altered function can lead to infertility and the development of germ cell tumors.^[^ ^65^ ^]^ SSC gene targets were *DPPA3*, *UTF1*, *TSPY4*, *DAZL*, and *STRA8*. *DPPA3* encodes an early germ cell transcription factor, while *UTF1* encodes a transcriptional repressor in quiescent SSCs whose dysregulation is associated with the loss of quiescent SSCs and impaired spermatogenesis.^[^ ^11^, ^66^ ^]^ *TSPY4* encodes an SSC‐specific protein, of which little is known as it has no mouse homolog but is predicted to function in chromatin organization and histone binding.^[^ ^67^ ^]^ Moreover, it correlates with a morphologically distinct subset of SSCs with highly condensed nuclei known as A_dark_ spermatogonia, believed to represent the quiescent SSC reserve.^[^ ^66^, ^67^, ^68^ ^]^ Meanwhile, *DAZL* encodes a broad translational regulator active in PGCs, SSCs, and spermatocytes, and its deletion is associated with spermatogenic arrest.^[^ ^11^, ^69^ ^]^ Lastly, *STRA8* encodes an SSC transcription factor that governs commitment to spermatogenesis, and whose altered function is associated with impaired initiation of spermatogenesis.^[^ ^19^, ^70^ ^]^

**Figure 10:**
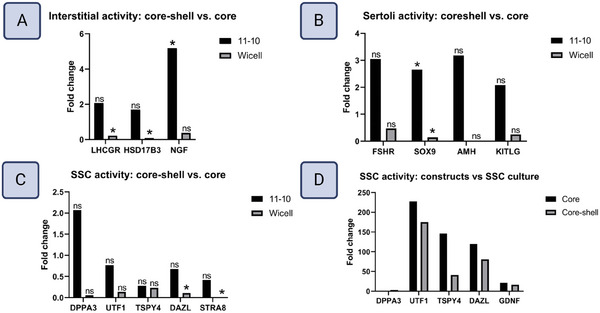
Changes in RNA expression of key genes regulating signaling factors and transcription factors, presented as the fold change of the core‐shell condition compared to the core‐only condition for A) Interstitial genes. B) Sertoli cell genes. C) SSC genes. N=3. Statistical significance calculated by Wilcoxon Exact test, α = 0.05, * = significant, n.s. = not significant. D) Fold change of SSC gene expression in the constructs compared to SSC cultures, showing the effects of co‐culture in 3‐D. N = 3 for print conditions, n = 1 for monolayer condition.

*LHCGR*, *HSD17B3*, and *NGF* transcription levels were higher in core‐shell constructs, with the increase in *NGF* statistically significant (**Figure** **10**A). Core‐shell constructs also exhibited higher expression of *SOX9*, *AMH*, *FSHR*, and *KITLG, with SOX9* statistically significant (Figure 10B). *UTF1*, *DAZL*, and *STRA8* were lower in core‐shell constructs, whereas *DPPA3* was slightly higher, with no changes being statistically significant (Figure 10C).

Conversely, incompletely differentiated controls exhibited a loss in expression of all targets in the core‐shell constructs compared to core‐only constructs, significantly so for *LHCGR*, *HSD17B3*, *SOX9*, *DAZL*, and *STRA8* (Figure 10A–C).

### 4.4 Core–Shell Constructs Induce Transcription of Canonical SSC Genes

Surprisingly, 7 days under print conditions increased transcript levels of canonical SSC marker genes in comparison to SSC cultures. *UTF1*, *DAZL, TSPY4, and DAZL* transcription were substantially higher in both core‐shell and core‐only constructs (Figure 10D), suggesting that Sertoli cell‐SSC cross‐talk may be key to promoting the expression of canonical SSC genes in vitro. To examine this further, the expression of *GDNF*, a Sertoli cell paracrine factor that regulates SSC state through binding to GFRA1, was compared between SSC cultures and the core‐shell constructs. Indeed, the constructs had greater levels of *GDNF* expression than SSC cultures (Figure 10D).

### 4.5 Core–Shell Constructs Functionalized with RA‐Releasing Microspheres Improve Function

Next, the core‐shell model was functionalized with biodegradable polycaprolactone RA‐MS, with the aim of mimicking in vivo‐like bioavailability of RA to improve function. Both retinol and RA degrade and isomerize rapidly upon exposure to light, heat, and oxygen, such that levels are quickly depleted under in vitro conditions.^[^ ^12^ ^]^ Conversely, RA‐MS can serve as local reservoirs providing continuous release of fresh RA.^[^ ^71^ ^]^

**Figure 11:**
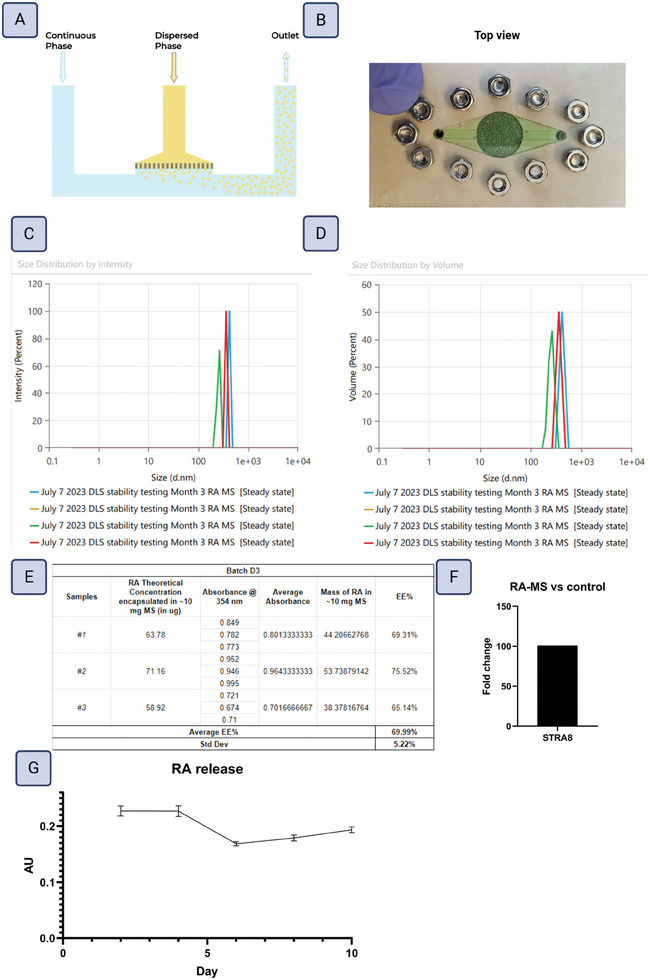
RA Microsphere generation and characterization. A) Schematic showing the step‐emulsification technique employed by the microsphere generator. A dispersed phase meets a continuous (aqueous) phase at a step where the dispersed phase is squeezed through a confined area. Once the dispersed phase passes through the step, a change in Laplace pressure causes the droplets to break up, generating microspheres. B) Top view of the microsphere generator. C,D) Size and uniformity characterization by two different measures, intensity and volume, using DLS, n = 4. E) Encapsulation efficiency of RA using spectrophotometry, n = 3. F) Effect of RA‐MS on RNA expression of *STRA8* immediately post‐printing. Data presented as the fold change of RA‐MS constructs compared to constructs without RA‐MS. N = 1. G) RA release profile over 10 days, measured by absorbance spectrophotometry and presented as the mean absorbance with standard deviation, n = 3.

RA was encapsulated into PCL microspheres using a microfluidic device and characterized for encapsulation efficiency, size distribution, and biological effect on SSCs. The microfluidic device was based on the step‐emulsification technique for droplet generation in which a dispersed phase meets a continuous (aqueous) phase at a step where the dispersed phase is squeezed through a confined area. Once the dispersed phase passes through the step, a change in Laplace pressure causes the droplets to break up, generating microspheres (**Figure** **11**A,B). Microsphere size and heterogeneity was determined by DLS and determined to be 5.282 ± 0.3159 µm with a polydispersity index of 1 (Figure 11C,D). Encapsulation efficiency was determined by spectrophotometry to be 69.99% ± 5.22% (11E). RA‐MS biological activity was confirmed by measuring *STRA8*, a direct target of RA activation in SSCs, in core‐shell constructs with RA‐MS compared to without immediately after printing (no media exposure)^[^ ^72^ ^]^ Core‐shell constructs functionalized with RA‐MS upregulated *STRA8* expression by 101‐fold compared to constructs without RA‐MS (Figure 11F). The release profile of RA from the microspheres under in vitro conditions was determined by monitoring RA levels by absorbance spectroscopy in PBS at 34 °C and 5% CO_2_ every 2 days over 10 days (Figure 11G). Results showed a slight burst release over the first 4 days before settling into a slower release.

**Figure 12:**
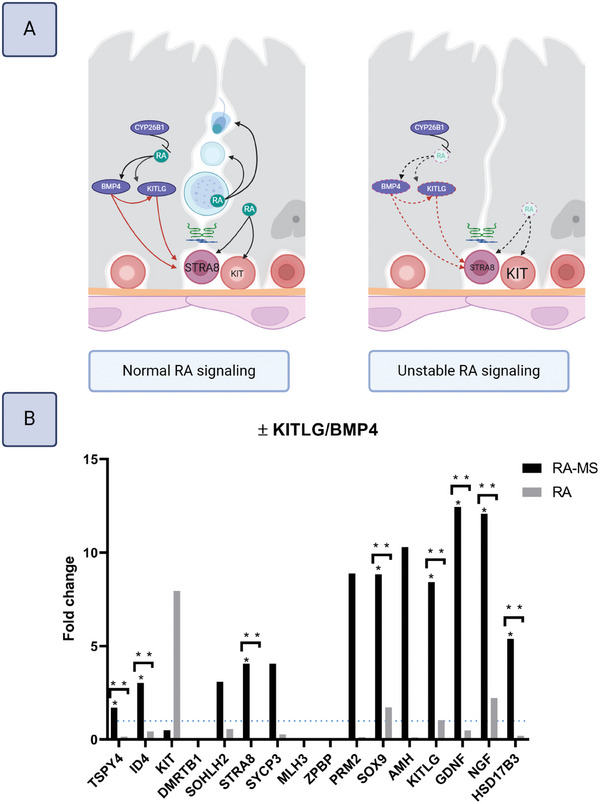
RA supplementation experimental schematic. A) Schematic showing RA activity in the adult testis (cross‐section of 2 Sertoli cells associating with multiple stages of differentiating germ cells). RA is generated and secreted by both Sertoli cells and germ cells, while the RA‐degrading enzyme CYP26B1 is secreted by Sertoli cells. Both are used to control local bioavailability of RA. Upon activation by RA, SSCs upregulate *STRA8* and *KIT* in preparation for spermatogenesis. RA acts on Sertoli cells to increase local KITLG and BMP4 production to support this action. BMP4 acts in an autocrine manner to further increase production of KITLG, resulting potentiated KITLG levels. B) Graph of core‐shell constructs with or without RA‐MS in the cores. Those without RA‐MS were supplemented with RA. The graph shows the effect of BMP4/KITLG supplementation on gene transcription. N = 3. Statistical significance calculated by Wilcoxon Exact test, α = 0.05. * indicates a significant difference between ±BMP4/KITLG conditions, ** indicates a significant difference between RA and RA‐MS conditions.

In vivo, RA triggers the major steps of spermatogenesis, and its bioavailability is controlled locally by cell secretion of degradative enzymes and conversion of retinol to RA (**Figure** **12**A).^[^ ^73^ ^]^ The Sertoli cell factors BMP4 and KITLG act cooperatively with RA and are required to initiate spermatogenesis. In mice it was determined that despite other sources of KITLG in the testis, Sertoli cell *KITLG* was necessary for spermatogenesis.^[^ ^74^ ^]^ Moreover, *KITLG* in Sertoli cells is observed to increase during prepuberty through the development of a heightened sensitivity to FSH. In mice, RA stimulates *KIT* and *STRA8* expression in SSCs, and *BMP4* and *KITLG* expression in Sertoli cells. KITLG binds to KIT on SSCs and is necessary for the initiation of spermatogenesis, while BMP4 further upregulates *KIT* and *STRA8*.^[^ ^75^ ^]^ Evidence points toward a similarly cooperative action between BMP4, KITLG, and RA in humans. Human SSCs express *KIT*, *BMPR2*, and *STRA8* during the initial stages of spermatogenesis, while *KITLG* expression in Sertoli cells increases in response to BMP4 stimulation.^[^ ^19^, ^64^, ^76^ ^]^ Moreover, *BMP4* and *KITLG* are abnormally low in men with a form of spermatogenic arrest known as non‐obstructive azoospermia (NOA), where Sertoli cells are characterized as immature.^[^ ^77^ ^]^

It was hypothesized that the addition of KITLG and BMP4 to culture medium should potentiate *KITLG* and *STRA8* activity through cooperation with RA signaling if it is stable. Core‐shell constructs were generated with or without RA‐MS mixed into the core bioink and cultured for 7 days, with or without BMP4 and KITLG supplemented into the medium. Those without RA‐MS were supplemented with free RA every 2 days. Under RA‐MS signaling, expression was significantly potentiated, indicating a cooperative action with BMP4 and KITLG, whereas under free RA signaling expression was slightly lost in the presence of BMP4 and KITLG (Figure 12B). Moreover, *KIT* was notably increased, suggestive of dysfunction. Ultimately, expression of *STRA8* and *KITLG* was significantly greater in the RA‐MS constructs by day 7 compared to those with free RA supplementation. Markers for later stages of spermatogenesis, including differentiating spermatogonia (*DMRTB1*, *SOHLH2*), meiotic spermatocytes (*SYCP3*, *MLH3*), and spermatids (*ZPBP*, *PRM2*), were investigated as well to ascertain if the prepubertal model was capable of initiating spermatogenesis at this stage. *SOHLH2*, *SYCP3*, and *PRM2* transcripts were lost while the rest were undetected. To observe additional widespread effects, the SSC markers (*TSPY4*, *ID4*) and somatic markers (*SOX9*, *AMH*, *GDNF*, *NGF*, *HSD17B3*) were also investigated. As with *STRA8* and *KITLG*, all increased significantly under RA‐MS signaling and were slightly lost under free RA signaling.

### 4.6 HiPSC‐Derived Sertoli Cell Constructs Mature and Express Tight Junctions

**Figure 13:**
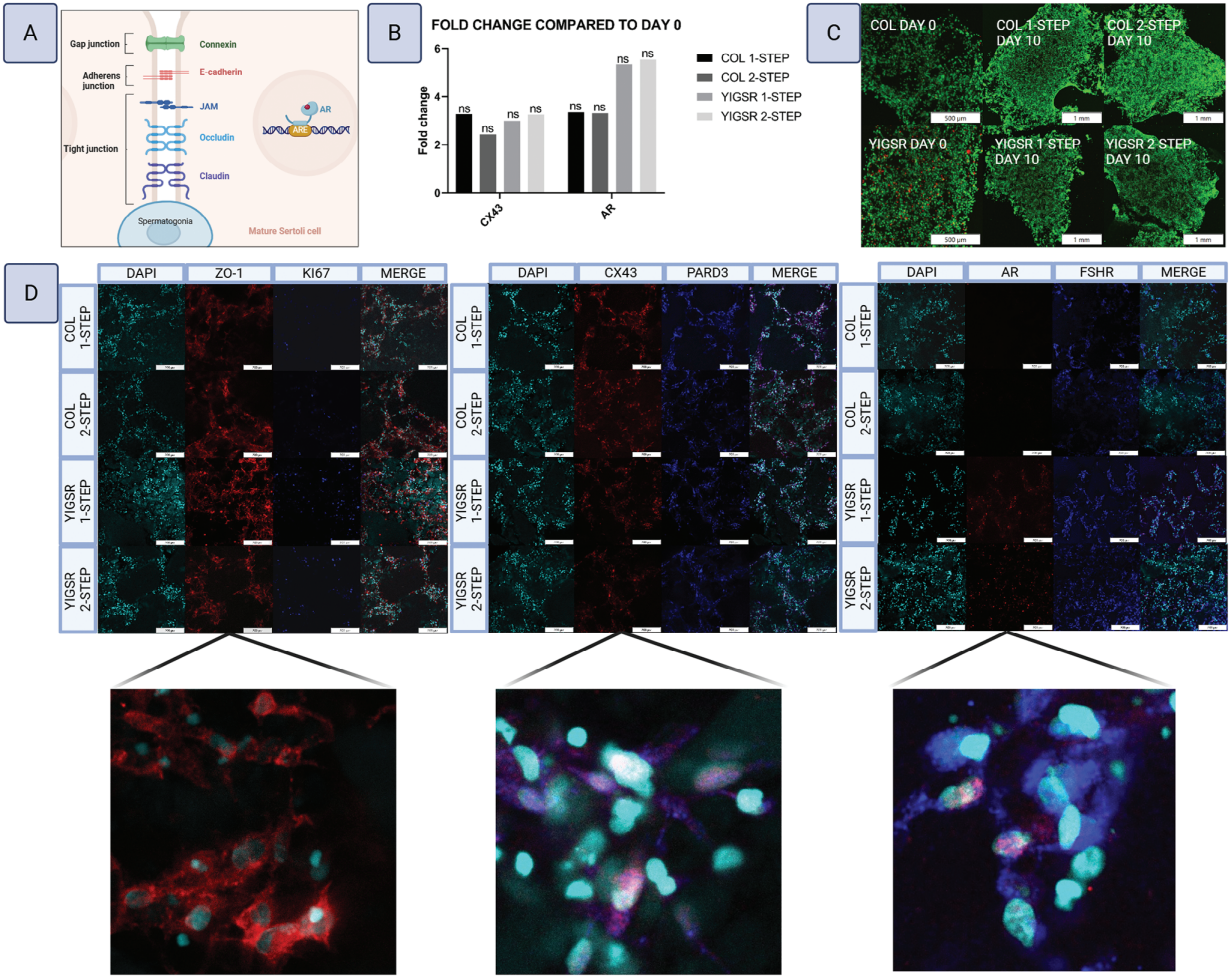
HiPSC‐derived Sertoli cell maturation. Results of 10 days in culture under conditions mimicking puberty. A) AR is a nuclear receptor that mediates Sertoli cell maturation by binding to testosterone to mediate its action on transcription through androgen response elements (AREs). It regulates the expression and organization of tight junctions including CX43, which is a second essential regulator for their maturation. B) Gene expression for *CX43* and *AR*. N=3, ns = not significant. C) Live/dead staining. D) Immunostaining for markers of maturation. Magnified boxes show localization of targets.

An important consideration toward the utility of this model lies in the capacity of hiPSC‐derived Sertoli cells to mature under the right conditions. In vivo, Sertoli cell maturation is triggered by testosterone during puberty. Their sensitivity to testosterone develops through the expression of the receptor AR, which first appears between the ages of 4.6–10.8 years old.^[^ ^55b^ ^]^ As Sertoli cells mature, they cease proliferation, develop tight junctions, and polarize (**Figure** **13**A).^[^ ^78^ ^]^ The gap junction CX43 is of particular importance in regulating this process, and it appears at the onset of puberty between the ages of 10‐8‐13.8 years old.^[^ ^55b^ ^]^ CX43‐knockout (KO) mice display a loss of tubule ultrastructure and an inability to mature or initiate spermatogenesis.^[^ ^79^ ^]^ To investigate this, constructs containing a high density of hiPSC‐Sertoli cells were printed and cultured under conditions mimicking the pubertal environment for 10 days. COL and YIGSR bioinks were compared to ascertain the importance of extracellular cues. Both were functionalized with RA‐MS. Two conditions were tested: a 1‐step program and a 2‐step program. The 1‐step program included supplementation with pubertal hormones testosterone, T3, and FSH, and lowered oxygen tension to 10% O_2_ since the mature testis is known to be oxygen‐deprived. The 2‐step program included a preliminary expansion step with morphogenic factors FGF2, FGF9 and activin A. Maturation in the hiPSC‐Sertoli cell constructs was examined by immunostaining for the proliferative marker KI67, the hormone receptors FSHR and AR, tight junctions ZO‐1 and CX43, and a polarity protein PARD3 (Figure 13C). All expressed ZO‐1, KI67, CX43, PARD3, and FSHR, but only the YIGSR bioink supported AR expression, confirming the importance of extracellular cues. AR expression was localized to the nuclei, indicating it was bound and active. Similarly, ZO‐1 was correctly localized to the cytoplasm and the surface membrane. Conversely, CX43 and PARD3 expression were mainly nuclear and weakly cytoplasmic, indicating they were not yet functional. Gene expression of *CX43* and *AR* was examined by RTqPCR and found upregulated after 10 days, with the YIGSR bioink showing the highest expression of *AR*, in line with immunostaining results (Figure 13B). Overall, the hiPSC‐Sertoli cells possessed the capacity to respond to pubertal conditions by initiating the expression of AR, ZO‐1, CX43, and PARD3.

## 5 Discussion

Human IVS progress is hindered by the scarcity of banked human prepubertal tissues donated to research.^[^ ^9^ ^]^ In IVS studies to date, detailed molecular analysis is likewise limited by the small size of the samples. Additionally, variability in tissue ages, ranging from 0.5–12 years old, is considered a factor contributing to the inconsistency of findings from one study to the next. HiPSCs represent a robust source of human testis cells, and due to their early developmental state, it was theorized that they could be used to model human prepubertal tissue to facilitate technological development in the pursuit of human IVS. Therefore, this work aimed to design an hiPSC‐derived prepubertal testis model.

Differentiating the major cell types of the testis from hiPSCs showed that efficient adoption of testis cell fates requires a mesoderm bias. The present study identified a commercially available hiPSC line (Cell Application iPS11‐10) with the capacity for efficient testicular cell fate derivation. In the future, employing early mesoderm‐priming steps such as the “incipient mesoderm‐like cell” (iMeLC) differentiation step could be investigated to improve testicular fate acquisition regardless of hiPSC line bias.^[^ ^26^, ^80^ ^]^

Single‐cell sequencing revealed the presence of subpopulations in the hiPSC‐derived testicular cell cultures characterized by distinct transcriptomic behavior. HiPSC‐Sertoli cell cultures separated into 2 subpopulations, one immature and morphogenic, and one in an intermediate state of maturation, sensitive to hormonal stimulation. HiPSC‐Leydig cell cultures similarly split into immature and maturing subpopulations. The immature group was characterized as immature due to thyroid hormone regulatory activity. Interestingly, the maturing group expressed the mature Leydig cell marker *HSD11B1* and exhibited cell‐cell coupling and macrophage homing activity, which is important for their function in vivo, where they form close associations with one another and intricate interdigitations with resident macrophages. Indeed, studies in mice show that Leydig cells do not develop normally without macrophages, and macrophages are observed to stimulate Leydig cell regeneration and steroidogenesis throughout adulthood.^[^ ^43^, ^81^ ^]^ Peritubular myoid cells were more homogeneous, consisting of a major subpopulation characterized by immature smooth muscle lacking *MYH11*, and by paracrine signaling, particularly *NGF*. A small progenitor subpopulation was present, and an even smaller *PAX8* subpopulation was noted. *PAX8* is a marker of rete testis progenitors and so it was hypothesized to be an off‐target cell fate. The SSC culture similarly exhibited a dominant subpopulation and three small subpopulations. The transcriptomic profile of the main subpopulation possessed numerous pre‐spermatogonia markers and was characterized by matrix building and proliferation. A small PGC subpopulation was seen, as was a small subpopulation expressing the SSC marker *TSPAN33*. The predominance of pre‐spermatogonia state activity may be explained by in vitro conditions, indicating that the culture system is still lacking many of the necessary cues found in the niche such as paracrine signal factors and ECM molecules. This may further explain the expression of connective tissue‐building genes and strong expression of the pre‐spermatogonia marker *COL1A2*, the major form of collagen deposited in the prepubertal niche.^[^ ^29^, ^46^ ^]^ Indeed, this form of upregulated tissue‐building expression has been reported to displace canonical SSC marker expression in long‐term primary human SSC cultures as well.^[^ ^34^ ^]^ To better understand where each hiPSC‐derived cell type was in its developmental trajectory they were each integrated with publicly available human testis datasets from various ages ranging from infant to pubertal to adult and subjected to pseudotime analysis. HiPSC‐Sertoli and hiPSC‐Leydig cells fell in with peripubertal samples, further supporting their composition as immature and maturing subpopulations. HiPSC‐peritubular myoid cells mapped as pre‐infant in the developmental trajectory, in line with their immature smooth muscle characterization. Intriguingly, hiPSC‐SSCs fell in with peripubertal samples, illustrating that, despite possessing some pre‐spermatogonia‐like activity, they were closer to a peripubertal state of development. This is further supported by their phenotype analysis confirming protein expression of adult SSC markers (*ZBTB16*, *GFRA1*, *GPR125*, *UCHL1*, *DDX4*, *ID4)*. Together, these observations suggest that hiPSC‐SSCs develop in a manner unique to in vitro culture conditions, wherein they adopt an overall peripubertal‐like phenotype but retain pre‐spermatogonia‐like behavior characterized by high proliferative and matrix‐building activity.

The unnatural transcriptomic activity observed in hiPSC‐SSC cultures illustrates a need to further optimize in vitro culture conditions; nevertheless, this study identified baseline conditions to support their growth, survival, and SSC‐like phenotype. Prior to this, SSC cultures relied on serum‐based conditions developed for neonatal mouse SSC,^[^ ^49^ ^]^ however, human SSCs were observed to lose their normal phenotype expression under these conditions, including expression associated with germ cell differentiation capacity.^[^ ^34^ ^]^ Therefore, hypothesizing that more human‐mimetic conditions would better support human SSCs in vitro, the mouse basal medium was replaced with one that mimics the composition of human plasma, animal serum components with synthetic xeno‐free alternatives, and through a screening process a xeno‐free matrix composed of dextran‐conjugated starPEG and functional motifs was identified to support their growth. Interestingly, the matrix screen revealed a preference for fibronectin and vitronectin motifs in line with findings from mouse and porcine SSC attachment studies.^[^ ^82^ ^]^ A recent study on human adult SSCs showed that Akt pathway inhibition in the presence of FGF2 and GDNF prevented their differentiation and loss of quiescence in vitro. Therefore, the small molecule Akt pathway inhibitor MK2206 was investigated as a component of hiPSC‐SSC expansion conditions. Replacing the neonatal mouse factor cocktail of EGF+GDNF+FGF2 with the factor cocktail of MK2206+GDNF+FGF2 improved survival and expression of quiescent SSC markers, particularly for the marker SSEA4, which was lost under the mouse factor condition. SSEA4 is associated with a subset of human and rodent SSCs with high self‐renewal potential.^[^ ^29a^ ^]^ Moreover, it is found to be consistently expressed in human SSCs throughout development from the fetal to the adult state, where it appears to be critical in balancing SSC quiescent and self‐renewal activity.^[^ ^29f^ ^]^ During spermatogenesis, SSCs adopt 3 distinct early states comprised of 2 quiescent states and an active proliferative state. One quiescent state is considered the reserve pool while the second quiescent state is primed for self‐renewal and characterized by GFRA1 and SSEA4. The proliferative state is considered a critical node in the balance between SSC self‐renewal and differentiation as it either progresses further into a differentiating state or returns to the quiescent SSEA4/GFRA1 state to replenish the pool.^[^ ^19a^ ^]^ Loss of SSEA4 in the hiPSC‐SSC cultures alongside diminished expression of other quiescent markers indicated a loss of this critical state under neonatal mouse SSC growth factor conditions.

Prepubertal testis cytoarchitecture is defined by cord‐like structures composed of immature Sertoli cells and SSCs surrounded by interstitium containing microvasculature and early peritubular myoid and Leydig cells. In order to mimic in vivo‐like signaling activity, coaxial extrusion bioprinting was used to generate core‐shell constructs populated with hiPSC‐derived Sertoli cells and SSCs in the core and hiPSC‐derived Leydig and peritubular myoid cells in the shell to resemble prepubertal cord‐interstitium cytoarchitecture. HiPSC‐derived testicular cells in this configuration captured functional aspects of human prepubertal testis within 7 days. Cross‐talk between the interstitial and cord compartments of the model was indicative of early morphogenic testicular activity. Notably, upregulation of *SOX9* and *DPPA3*, two early regulators of Sertoli cells and SSCs,^[^ ^11^, ^23^, ^61^, ^66^ ^]^ as well as *NGF*, a growth factor critical to testis morphogenesis,^[^ ^45^, ^60^ ^]^ were observed in response to core‐shell cross‐talk.

Collagen IV and α‐laminin are key ECM components in the prepubertal testis cords.^[^ ^58^ ^]^ Indeed, a human prepubertal IVS study identified a correlation between disrupted deposition of α‐laminin and the loss of SSCs in vitro.^[^ ^58^ ^]^ The bioinks used in the core‐shell model were composed of bioinert cellulose functionalized with peptide motifs found in collagen and laminin proteins (COL and YIGSR), which were anticipated to activate ECM remodeling, and indeed, deposition of both collagen IV and α‐laminin by hiPSC‐derived testicular cells was observed within 7 days. Nevertheless, cells remained rounded at day 7 rather than adopting morphologies observed in vivo, implying that longer culture periods are necessary for adequate accumulation of native matrix to support their normal phenotype.^[^ ^83^ ^]^

For comparison, an ectoderm‐biased hiPSC line was differentiated in parallel and found capable of only partial induction into testicular lineages. Core‐shell constructs were created from these incompletely differentiated cells as an additional control and analyzed in parallel. The incompletely differentiated core‐shell model downregulated transcription of all target genes upon interaction between the shell and core compartments and failed to exhibit deposition of α‐laminin within the core compartments. Their dysfunction underscores the importance of efficient cell fate induction in hiPSC‐derived tissues.

The utility of the model was tested by comparing two methods of RA supplementation on the initiation of spermatogenesis. In vivo, spermatogenesis is asynchronous and overlapping, and this coordinated activity is regulated by RA signaling, generated and degraded locally by cellular enzymes.^[^ ^73^ ^]^ In vitro, free RA supplemented into medium is subject to extensive degradation and isomerization within min from standard fluorescent room lights or sunlight, incubator heat, and oxygen exposure.^[^ ^12^ ^]^ A second limitation to this approach lies in the inability of medium to diffuse the necessary distance into the tissues, and for RA this distance is further minimized by local degradative activity by interstitial cells. To maximize medium diffusion in rodent IVS systems, tissue fragments are sandwiched between a medium‐soaked agarose block and an oxygen‐permeable polydimethylsiloxane (PDMS) ceiling to spread them thin and flat.^[^ ^7^, ^84^ ^]^ Nevertheless, translating this system to porcine tissues has proven unsuccessful, resulting in tissue degeneration and loss of SSCs.^[^ ^8b^ ^]^ Human IVS studies have yielded only inconsistent reports regarding the effects of RA supplemented into medium to initiate spermatogenesis. One study found spermatogenesis was possible in the absence of RA but not with RA, while others reported no spermatogenesis with or without RA, and another found initiation only in the presence of RA.^[^ ^9b,c,f,g^ ^]^ It was hypothesized that a more stable and local RA action may be necessary to support human IVS. To this end, RA was encapsulated into the biodegradable polymer PCL and processed to form microspheres, which exhibited a steady release of RA. These microspheres were mixed into core‐shell constructs to promote localized, stable RA activity and compared with the activity of free RA from medium supplementation. It was hypothesized that the core‐shell constructs would possess an immature response to RA stimulation, failing to initiate spermatogenesis. Nevertheless, the supplementation of RA with RA‐cooperative factors BMP4 and KITLG could be expected to potentiate RA‐mediated expression of *STRA8* and *KITLG*, and so their supplementation was used as a means to interrogate RA‐dependent activity in the model (Figure 11B). Free RA signaling was insufficient to cooperate with BMP4 and KITLG to potentiate *KITLG* and *STRA8*, whereas the slow‐release of RA from local microspheres generated the expected response, resulting in significant potentiation of *KITLG* and *STRA8*. SSC markers (*TSPY4*, *ID4*) and other somatic markers (*SOX9*, *AMH*, *GDNF*, *NGF*, *HSD17B3*) were also significantly potentiated under RA‐MS signaling, whereas they were largely diminished under free RA signaling, illustrating that the effects of poor RA signaling disrupt functionality throughout the niche. Despite exhibiting a strong response to RA, the model was not capable of upregulating widespread spermatogenic expression. In particular, *DMRTB1* was undetected, which is of particular importance as this gene coordinates the transition of *STRA8*+ spermatogonia into spermatocytes by repressing spermatogonia genes such as *SOHLH2* and activating genes required for meiotic prophase.^[^ ^85^ ^]^ Its absence speaks to the immature state of the model. The inability of free RA signaling to cooperate with BMP4 and KITLG supplementation supports our theory that the rapid degradation of medium‐supplemented RA is likely a contributing factor to the dysfunction observed in human prepubertal IVS studies to date. Overall, these findings highlight the importance of local paracrine signaling in IVS systems, warranting investigation into other factor‐releasing microspheres to improve in vitro supplementation.

A consistent limitation observed amongst human IVS studies is the disorganization of cell–cell junctions in vitro.^[^ ^9a,b,d^ ^]^ Therefore, while the core‐shell geometry of the model is a step forward in promoting biomimetic cellular organization, physical interactions between cells, particularly the formation of cell‐cell junctions, are an important aspect to consider developing further. In vivo, Sertoli cell tight junctions develop at puberty, mediated by testosterone signaling, and this action is tightly coupled to their polarization and maturation. By culturing hiPSC‐Sertoli cells within constructs at high density under conditions mimicking the pubertal environment, including lowered oxygen tension and hormonal stimulation, it was possible to observe the activation of AR expression in the constructs along with tight junction formation of ZO‐1 and early disorganized expression of CX43, a later stage gap junction protein required for maturation, as well as PARD3, a polarity protein.^[^ ^55^, ^79^ ^]^ These findings confirm the capacity of the model to express AR and generate cell‐cell junctions and polarity proteins under the right conditions, and as such, the optimization of cell seeding density and maturation conditions warrants further exploration to promote their organization within the core‐shell constructs toward mature functionality. Intriguingly, a recent study on human intestinal epithelial cells has shown that it may be possible to uncouple the development of tight junctions and polarity from cell‐cell contact through genetic engineering. In that study, an inducible vector system was used to force the expression of *STRAD*, which activates the polarity gene *LKB1*, inducing rapid polarity including the generation of an apical brush border and the peripheral localization of tight junctions in the absence of cell‐cell contact.^[^ ^86^ ^]^ Similarly engineered hiPSC‐derived Sertoli cells could be an alternative route to explore as a means of promoting polarization and organizing tight junction formation.

A second consistent finding across human prepubertal IVS studies is the loss of SSCs and proliferative SSCs.^[^ ^9a–c,e–g^ ^]^ In fetal tissues, the use of GDNF+EGF+FGF2 was credited with supporting SSC survival and proliferation in vitro, but this did not translate to infant tissues, and GDNF was likewise found to have no effect on SSC proliferation in older prepubertal tissues.^[^ ^9^, ^87^ ^]^ There is a need to study the efficacy of GDNF supplementation to support SSC self‐renewal in the testicular microenvironment in vitro as well as other conditions that may improve SSC survival and proliferation.^[^ ^9c,e–g^ ^]^ Intriguingly, comparison of the core‐shell model to SSC cultures revealed a substantial increase in SSC expression of key regulatory factors, including *UTF1*, *TSPY4*, and *DAZL*.^[^ ^66^, ^67^, ^68^, ^69^ ^]^ This difference is likely attributed to Sertoli cell‐SSC paracrine interactions, perhaps including GDNF‐GFRA1 signaling. Indeed, GFRA1 expression was confirmed in hiPSC‐SSC cultures while *GDNF* expression was found upregulated within the constructs compared to SSC culture. This suggests that supplementation methods need to be improved to match that of paracrine signaling, whether it is through specific combinations of factors, their concentrations, or perhaps their spatial localization within ECM, which is known to couple with factors to promote signaling.^[^ ^88^ ^]^

The vascular component of the testicular niche is understood to contribute to spermatogenesis; however, it was excluded in this model for the sake of simplicity since it has been demonstrated expendable for IVS. In a study using rodents, IVS was compared between isolated tubules versus whole tissue fragments ‐ which contained the major proportion of Leydig cells as well as the vasculature component including endothelial cells, pericytes, macrophages, and stem cells – and found to be reduced in the isolated tubules, but still active up to the elongating spermatid stage. Nevertheless, further development of the core‐shell model should seek to improve complexity and functionality by incorporating a vascular component. The primary challenge will be to generate testis‐specific vascular phenotypes. Examples of phenotypes identified as unique to testicular vasculature include endothelial cells which generate SSC maintenance factors, and two distinct resident macrophage populations with osteoclast and microglia‐like properties.^[^ ^89^ ^]^ Following lineage acquisition from a stem cell state, the adoption of tissue‐specific phenotypes in vasculature are believed to require exposure to extrinsic factors in the relevant niche during development.^[^ ^90^ ^]^ Indeed, hiPSC‐derived endothelial cells lack tissue‐specific phenotypes and are only roughly defined as either metabolically active (CLDN5+), immune‐responsive (APLNR+), arterial (GJA5+), or activated (ESM1+), while hiPSC‐derived macrophages appear most similar to the erythroid myeloid progenitors – an immature lineage with capacity to give rise to tissue‐resident macrophages and microglia.^[^ ^90^, ^91^ ^]^ Incorporation of hiPSC‐derived vascular cell lineages to the core‐shell model could, therefore be investigated as a means to generate testicular vasculature and improve the complexity of the model. An alternative approach may be the incorporation of adipose‐derived microvessels, available via liposuction procedure and subsequent adipose stromal vascular fraction (ASVF) preparations. Like hiPSCs, these can be autologously harvested from patients but are also available off‐the‐shelf (Advanced Solutions). They contain the entire milieu of microvascular cell types including endothelial, pericyte, macrophage, and progenitor, with intact native microvessel cytoarchitecture.^[^ ^92^ ^]^ These small microvessel fragments are used as seeds capable of giving rise to neovasculature in vitro. Importantly, they exhibit considerable organotypic plasticity when present within a specific tissue environment. For example, when combined with astrocytes in 3‐D culture, they adopt brain endothelial markers, and vessel permeability approaches blood‐brain‐barrier levels.^[^ ^93^ ^]^

Since hiPSCs can be derived from patients, the model may also have application in the study of patient‐specific forms of infertility such as NOA, including idiopathic or those with known genetic factors.^[^ ^13^, ^94^ ^]^ Organotypic modeling approaches of male infertility are ultimately limited by an inability to overcome inherent functional deficits such as those found in NOA, and therefore an hiPSC approach that enables cell type isolation and corrective intervention prior to 3‐D culture may confer an advantage. For example, hiPSCs created from infertile men with Y chromosome azoospermia factor (AZF) region deletions show diminished ability to differentiate into germ‐like cells upon transplant into mouse seminiferous tubules.^[^ ^94d^ ^]^ Similarly, hiPSC lines derived from patients with idiopathic NOA display a compromised ability to differentiate into germ‐like cells using molecular differentiation protocols.^[^ ^94a^ ^]^ The nature of the hiPSC core‐shell model provides a means to control the components of the early testicular microenvironment and introduce gene editing techniques to interrogate such inherent functional deficits.

## 6 Conclusion

Overall, findings from this study suggest that hiPSC‐derived testicular cells can be used to effectively model prepubertal human testicular tissue. Their derivation from hiPSCs gives rise to immature testicular populations, confirmed through immunostaining and transcriptome profiling. The bicompartmental 3‐D model promotes the immature activity of hiPSC‐derived testis cells through cross‐talk between the core and shell, while the juxtaposition of SSCs with Sertoli cells in the cores promotes the maintenance of SSCs. HiPSC‐derived Sertoli cells display the capacity for maturation through AR, tight junction, and polarity protein expression under appropriate conditions. Finally, bioink functionalization with microsphere‐encapsulated RA improves germ and somatic cell transcriptional activity, suggesting the utility of this approach as a means for promoting in vivo‐like factor support over standard media supplementation.

## Conflict of Interest

Stephanie Willerth is the CEO of Axolotl Biosciences. Ryan Flannigan is the recipient of an Honorarium from Ferring, an Educational Grant from Boston Scientific, and is the Co‐Founder of Teumo Health Technologies Inc. All other authors have no conflicts of interest.

## Supporting information



Supporting Information

Supporting Information

Supporting Information

## Data Availability

The data that support the findings of this study are available in the supplementary material of this article.
