# Supplementary material for: 3D Bioprinted Coaxial Testis Model Using Human Induced Pluripotent Stem Cells:A Step Toward Bicompartmental Cytoarchitecture and Functionalization
Source: Adv Healthc Mater. 2025 Feb 16;14(10):2402606. doi: 10.1002/adhm.202402606 (PMC12004438; doi:10.1002/adhm.202402606)
Supplement: Supplementary file 1 — Supporting Information [file ADHM-14-0-s002.docx]

**Supporting Information**

***RA microsphere generator assembly***

SolidWorks platform (Solidworks®, USA) was used to design the microsphere generator. The designs were modelled as an assembly in SolidWorks, and the layers were manufactured using a computer numerical control (CNC) machine (MDA precision®) and laser cutter (BossLaser®). The G-code required for the CNC operation was developed using the SolidWorks add-in (CAM) The laser cutter was used to cut the desired designs for assembly purposes. Supplemental figure 1 shows the breakdown of the generator’s components and view of the assembled generator. The cartridge consists of a porous stainless-steel disc, known as a sintered filter (McMaster-Carr, 9446T33), embedded within a stack of three machined layers and supported with 1.5—and 2.5-mm thick laser cut gaskets. The top polyether ether ketone (PEEK, McMaster-Carr, 9089K123) layer includes the device’s inlet and outlet ports. The middle layer is made of PEEK and contains through holes for the inlet and outlet ports, the disc housing on one side and the microfluidic channel on the other. The third layer is a transparent polymethyl methacrylate (PMMA, McMaster-Carr, 4615T63) layer coated with fluorinated ethylene propylene (FEP, McMaster-Carr, 5805T11) to protect the PMMA from DCM. The whole cartridge was assembled using M4 screws (McMaster-Carr, 91828A231/91292A122).


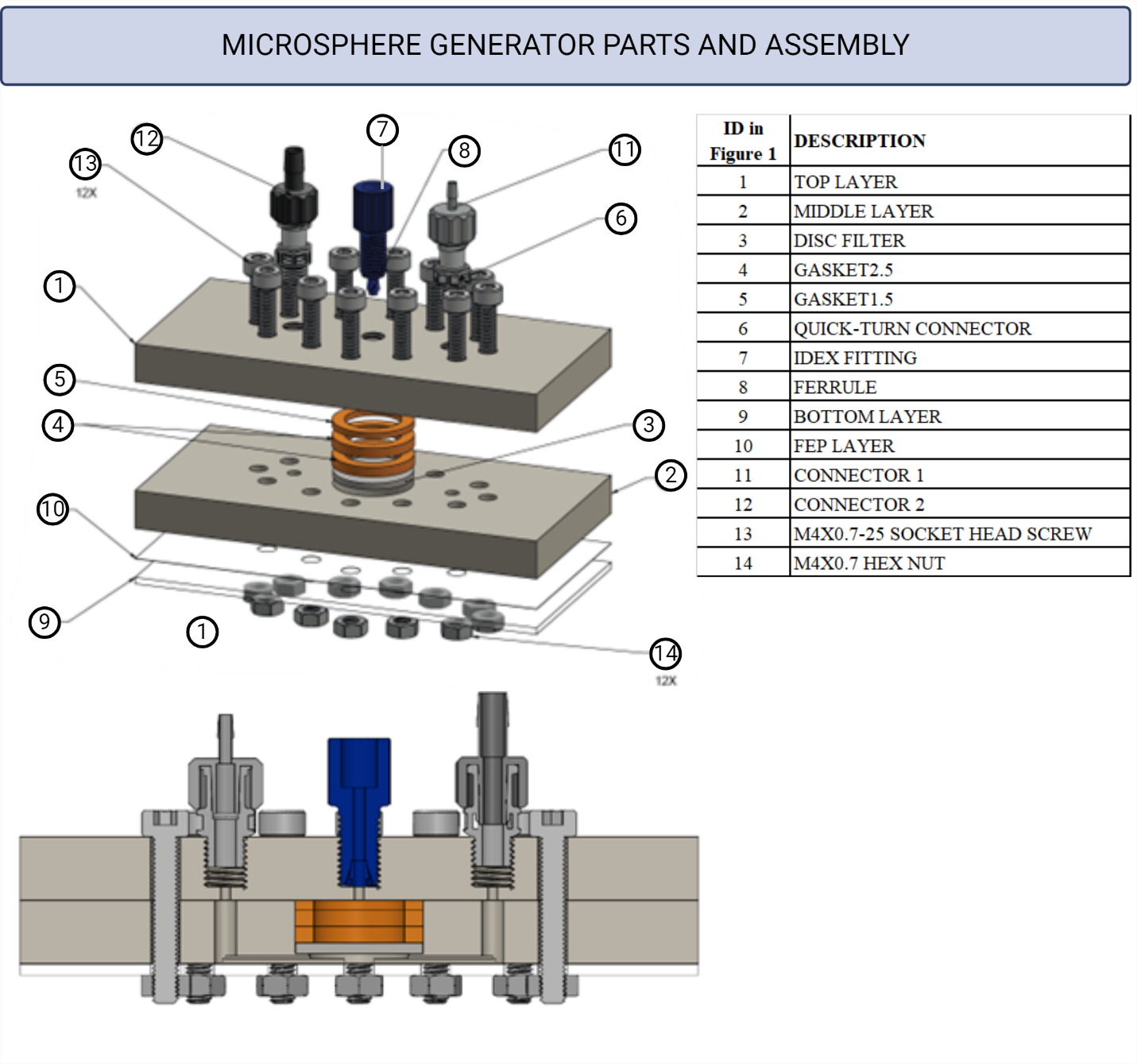
 *Supplemental file 1, Figure 1*. Exploded assembly and cross-sectional view of the microsphere generator.

***RA-MS prints additional characterization***

The core-shell geometry of the prints was visually confirmed using fluorescent cell tracers while viability was confirmed by live/dead staining (supplemental figure 2A-B). Following 7 days culture, cell-matrix interactions were confirmed by immunostaining for cytoskeletal filaments vimentin and β-tubulin (supplemental figure 2C). The addition of RA or RA-MS did not appear to impact viability or cell-matrix interactions.


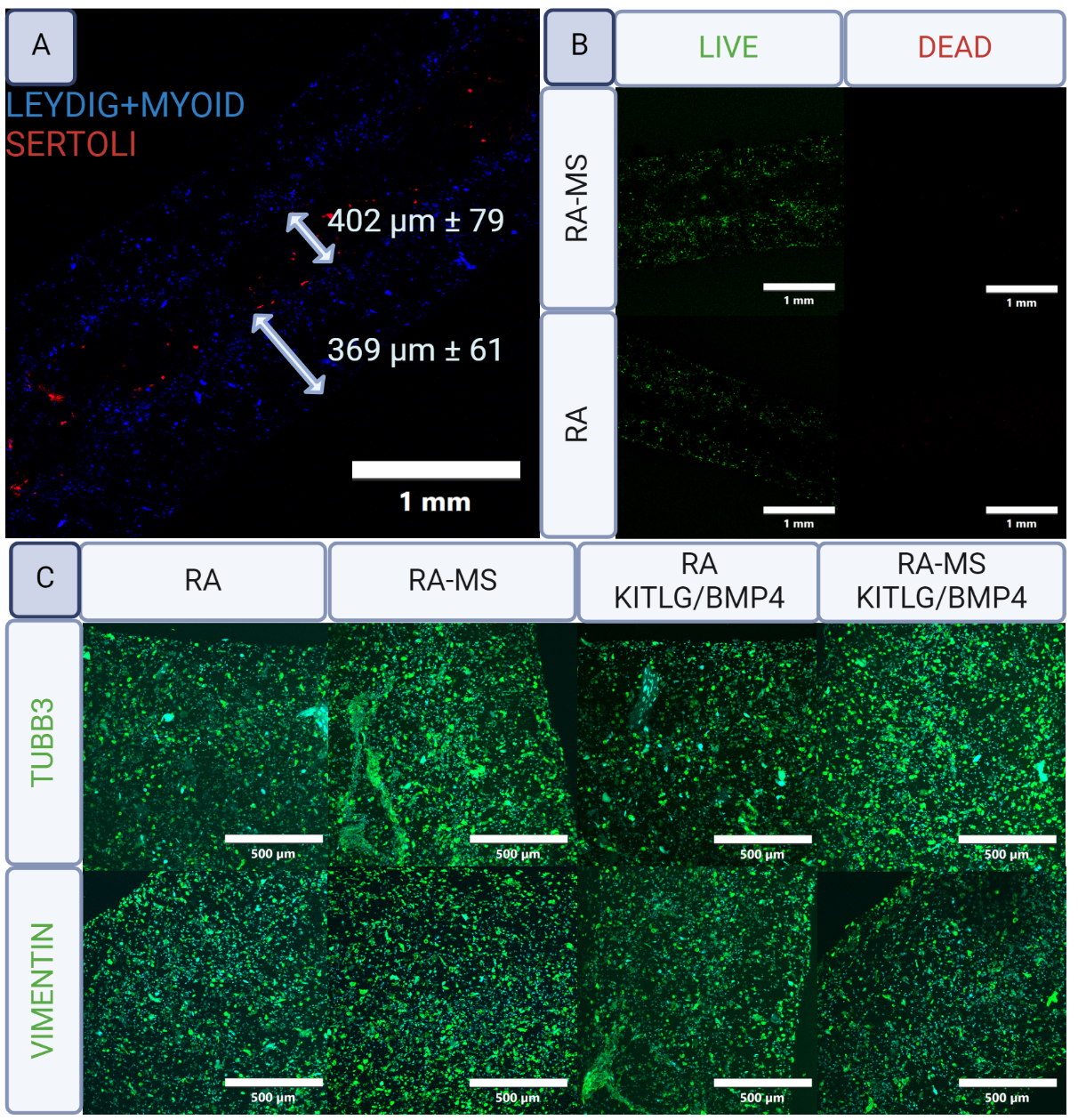


*Supplemental file 1, figure 2*. RA-MS prints characterization. A) Core-shell dimensions, visualized by fluorescent cell tracers and measured. Red in the cores, and blue in the shells. N=70. B) Live/dead staining immediately following printing. N=3. C) Immunostaining for cytoskeletal filaments following 7 days culture. N=3.
