# Supplementary material for: 3D Bioprinted Coaxial Testis Model Using Human Induced Pluripotent Stem Cells:A Step Toward Bicompartmental Cytoarchitecture and Functionalization
Source: Adv Healthc Mater. 2025 Feb 16;14(10):2402606. doi: 10.1002/adhm.202402606 (PMC12004438; doi:10.1002/adhm.202402606)
Supplement: Supplementary file 2 — Supporting Information [file ADHM-14-0-s003.docx]

***PIPseq additional plots***


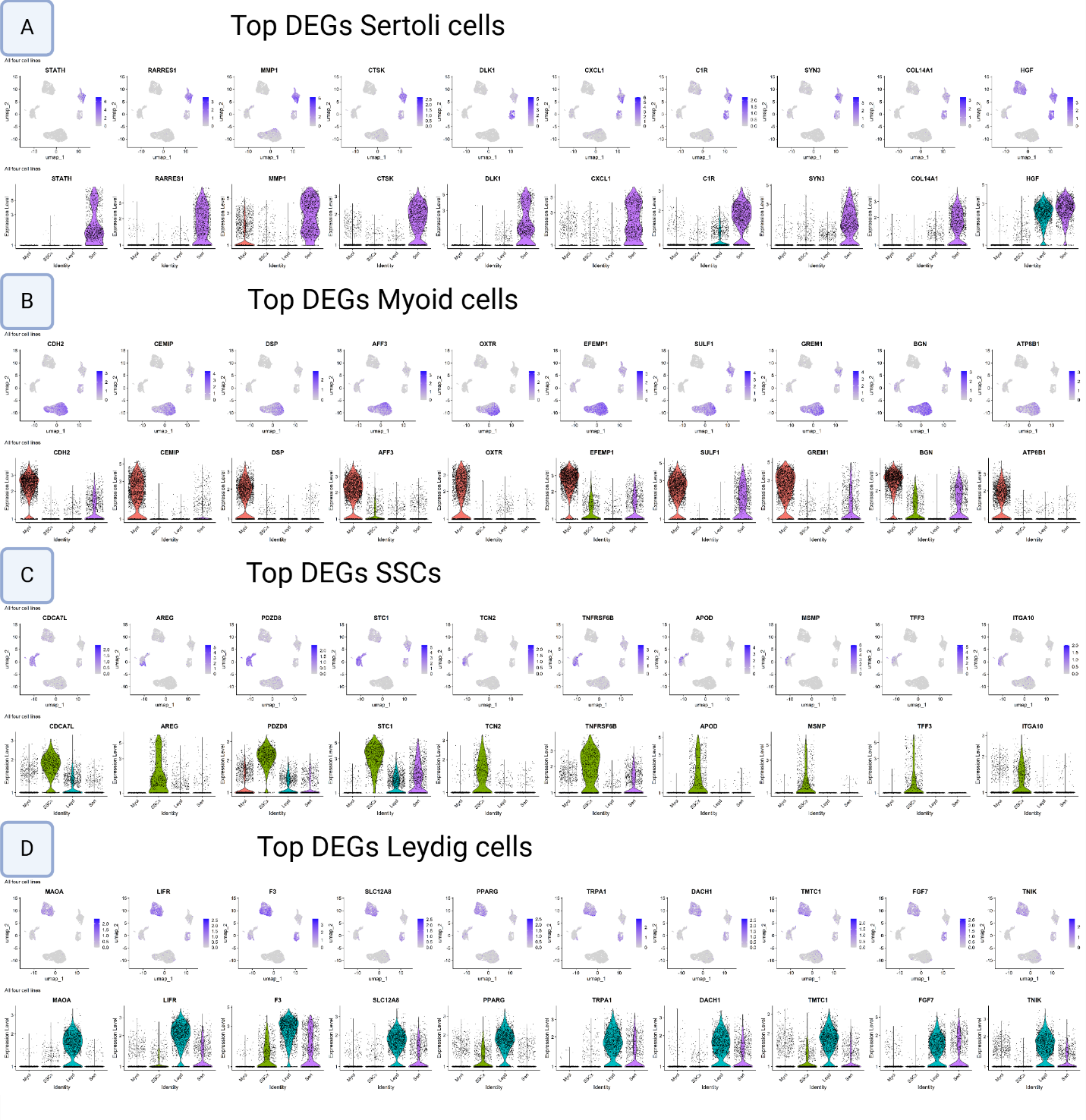


*Supplemental file 2, Figure 1.* Feature plots and violin plots for the top DEGs in the A) hiPSC-Sertoli cell population, B) hiPSC-peritubular myoid cell population, C) hiPSC-SSC population, D) hiPSC-Leydig cell population.


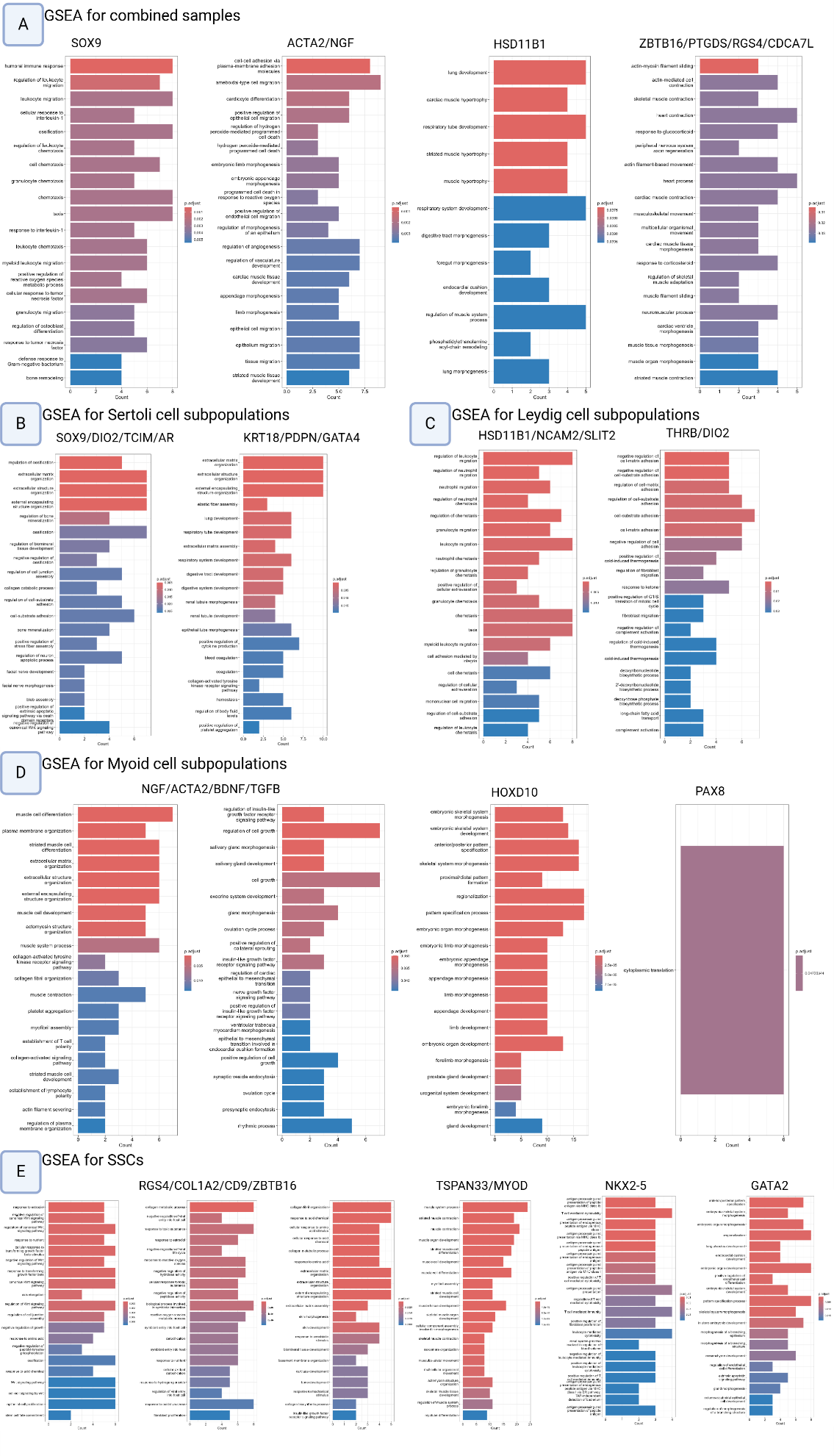


*Supplemental file 2, Figure 2.* GSEA bar plots from the top DEGs in the A) combined samples, B) hiPSC-Sertoli cell subpopulations, C) hiPSC-Leydig cell subpopulations, D) hiPSC-peritubular myoid cells subpopulations, and D) hiPSC-SSC subpopulations.


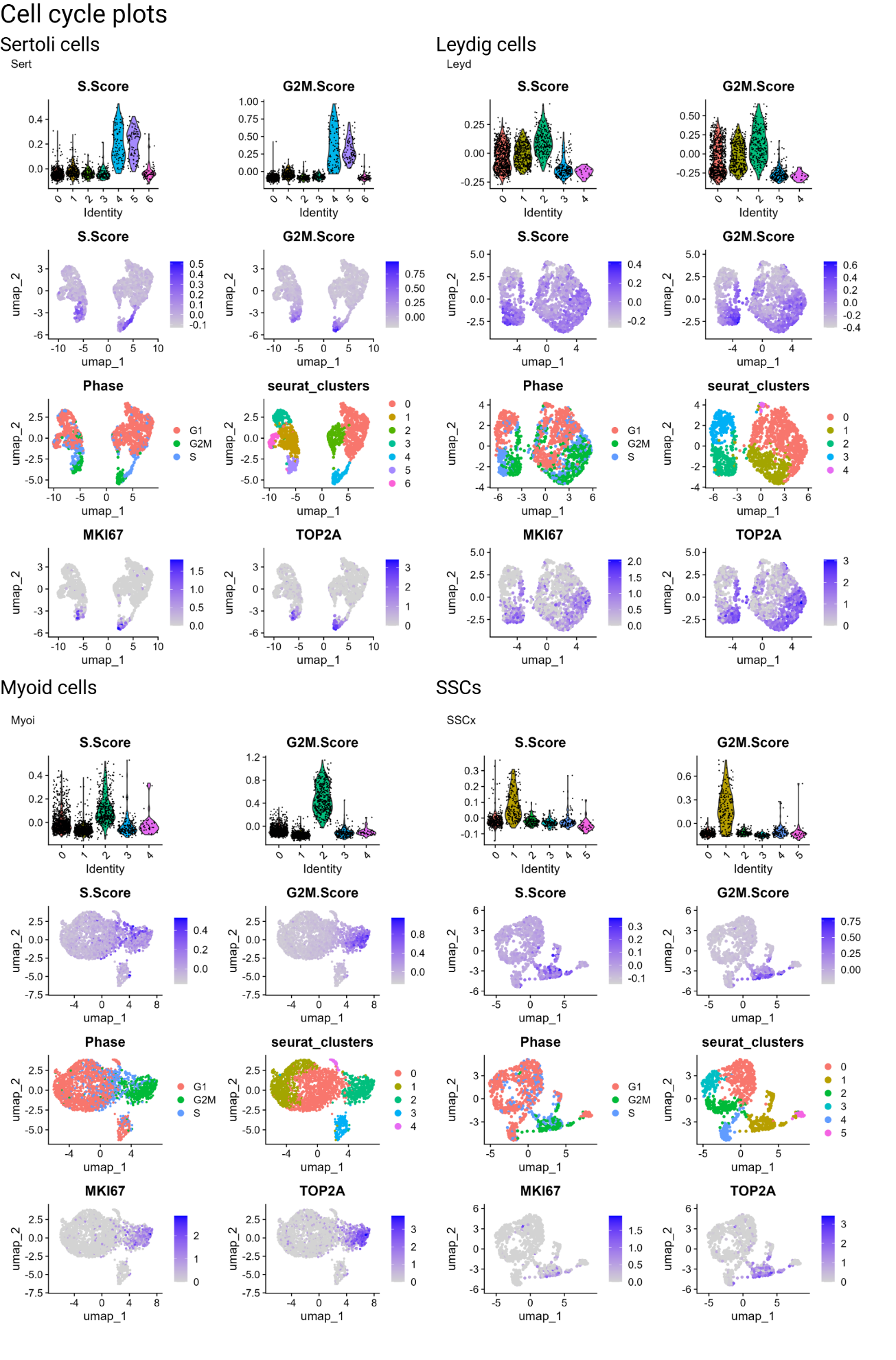


*Supplemental file 2, Figure 3.* Cell cycle plots.


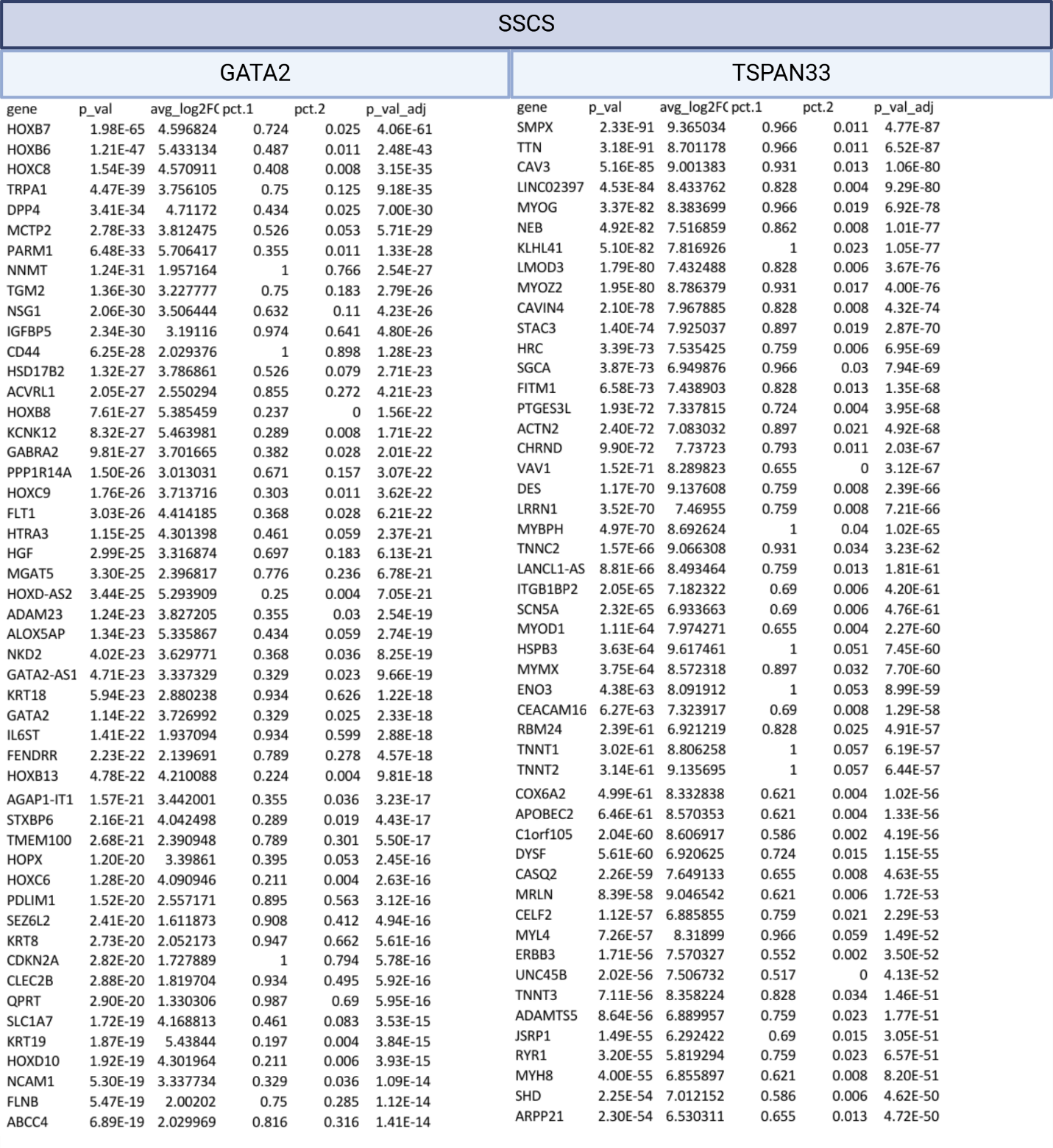


*Supplemental file 2, Figure 4.* Top DEGs for hiPSC-SSC subpopulations.


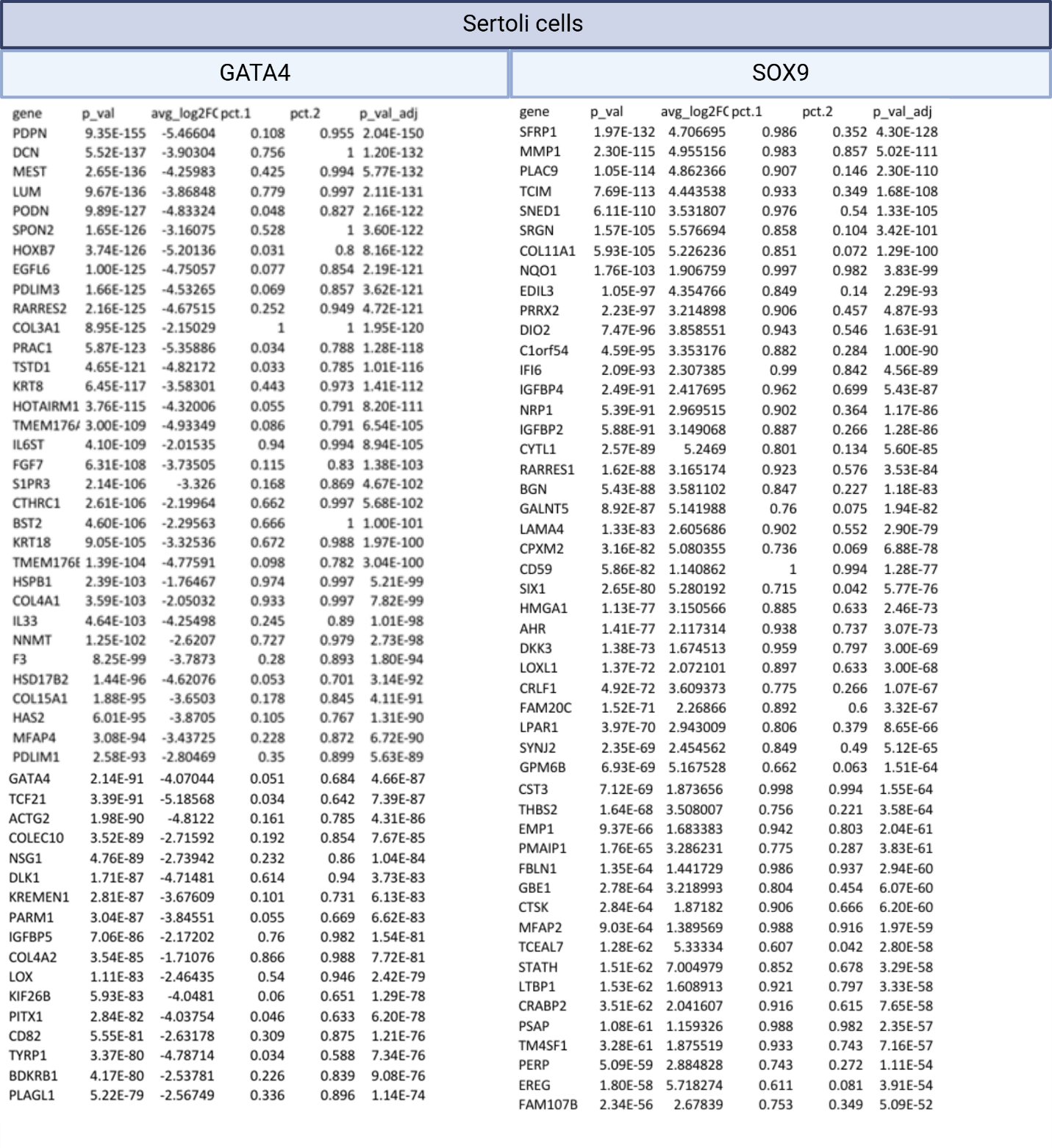


*Supplemental file 2, Figure 5.* Top DEGs for hiPSC-Sertoli cell subpopulations.


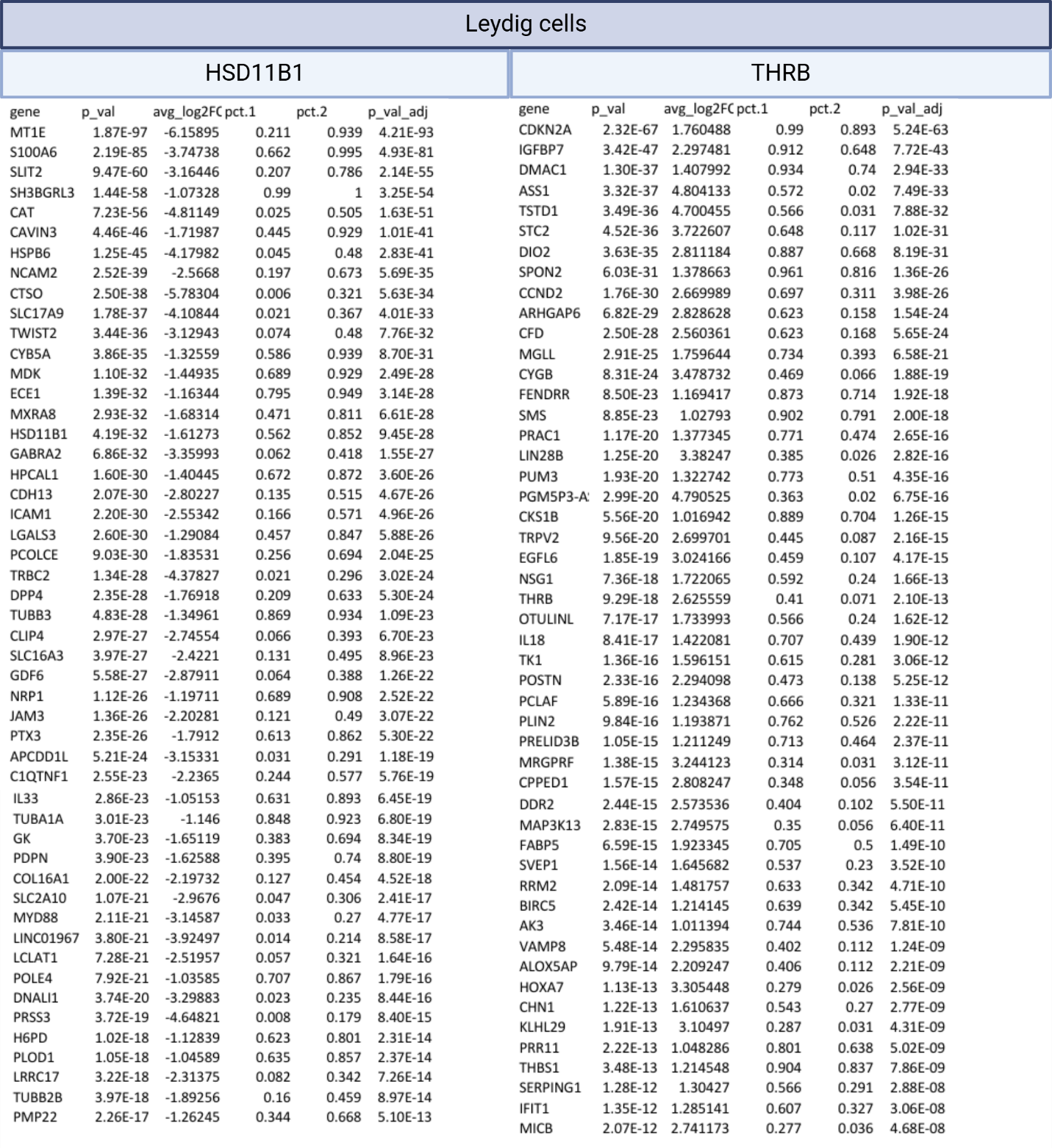


*Supplemental file 2, Figure 6.* Top DEGs for hiPSC-Leydig cell subpopulations.


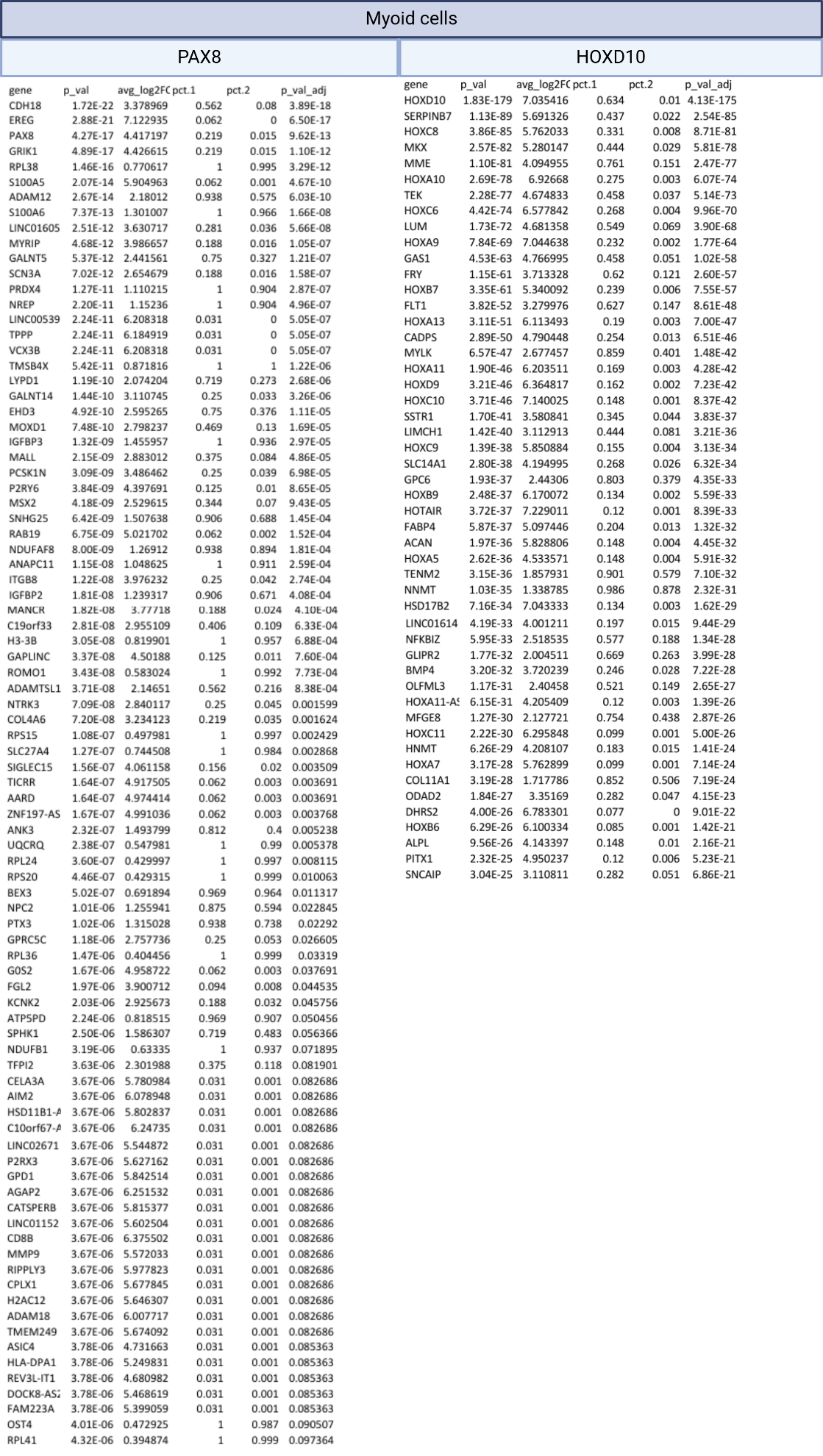


*Supplemental file 2, Figure 7.* Top DEGs for hiPSC-myoid cell subpopulations.
