# Supplementary material for: 3D Bioprinted Coaxial Testis Model Using Human Induced Pluripotent Stem Cells:A Step Toward Bicompartmental Cytoarchitecture and Functionalization
Source: Adv Healthc Mater. 2025 Feb 16;14(10):2402606. doi: 10.1002/adhm.202402606 (PMC12004438; doi:10.1002/adhm.202402606)
Supplement: Supplementary file 3 — Supporting Information [file ADHM-14-0-s001.docx]

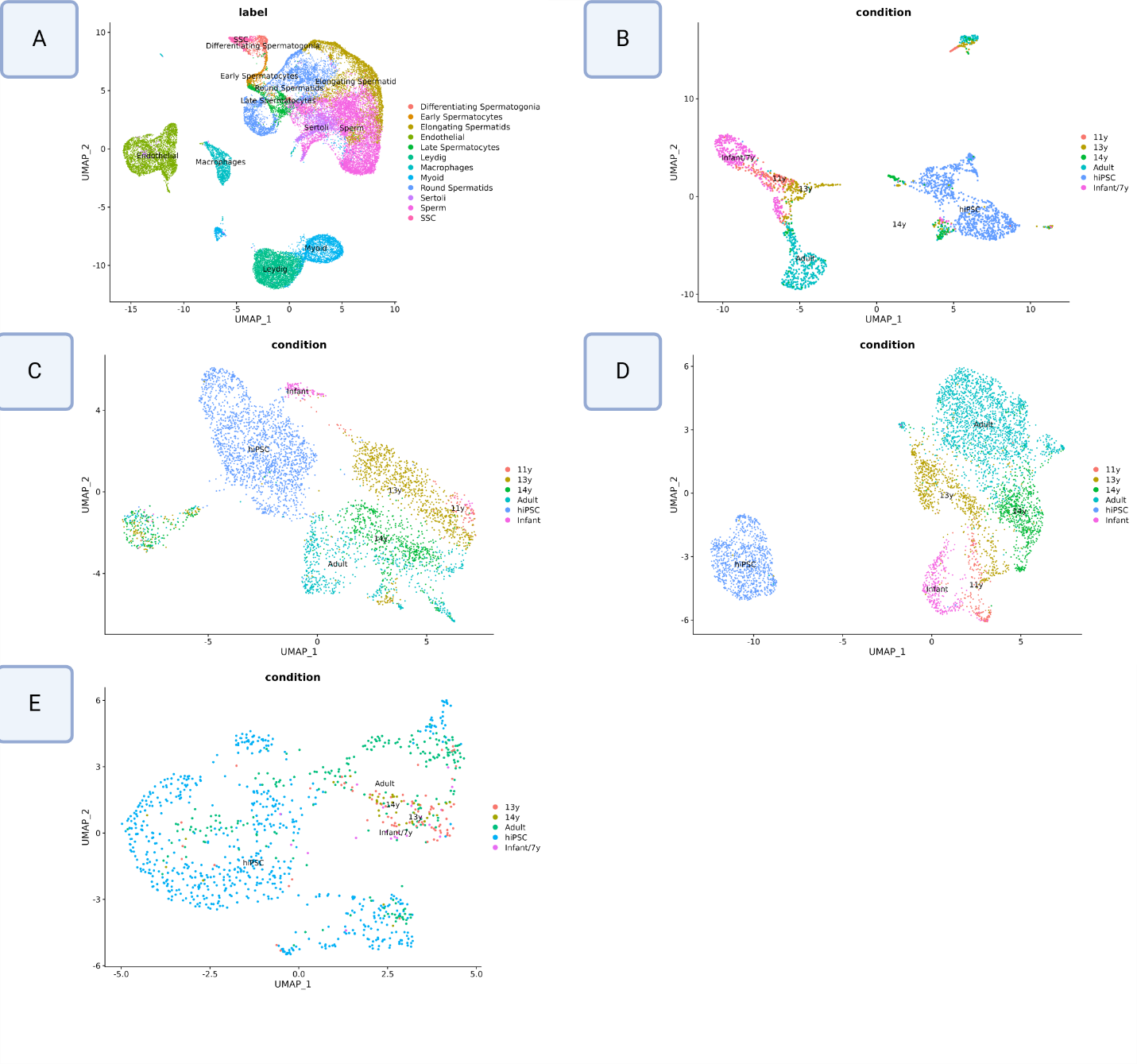


*Supporting file 3, Figure 1*. Cell type clustering (UMAPs) following integration with public human testis single cell databases. A) All *in vivo* human testis samples merged and annotated. B) *In vivo* primary Sertoli cells integrated with hiPSC-Sertoli cells. C)*In vivo* primary peritubular myoid cells integrated with hiPSC-peritubular myoid cells. D) *In vivo* primary Leydig cells integrated with hiPSC-Leydig cells. E) *In vivo* primary SSCs integrated with hiPSC-SSCs.


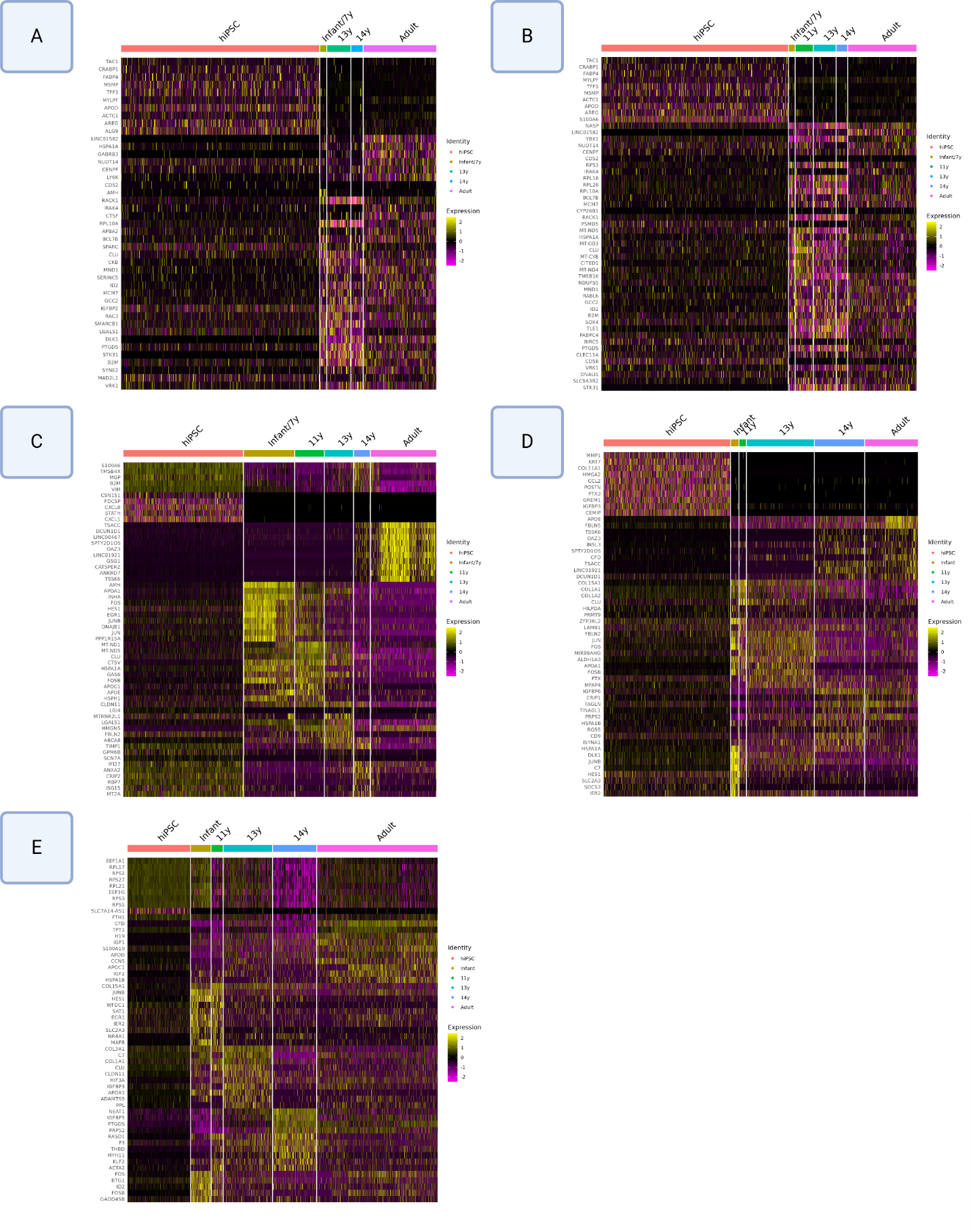


*Supporting file 3, Figure 2*. Heatmaps showing DEGs between the integrated datasets. A) SSCs. B) SSCs including the 11-year-old sample. C) Sertoli cells. D) Peritubular myoid cells. E) Leydig cells.


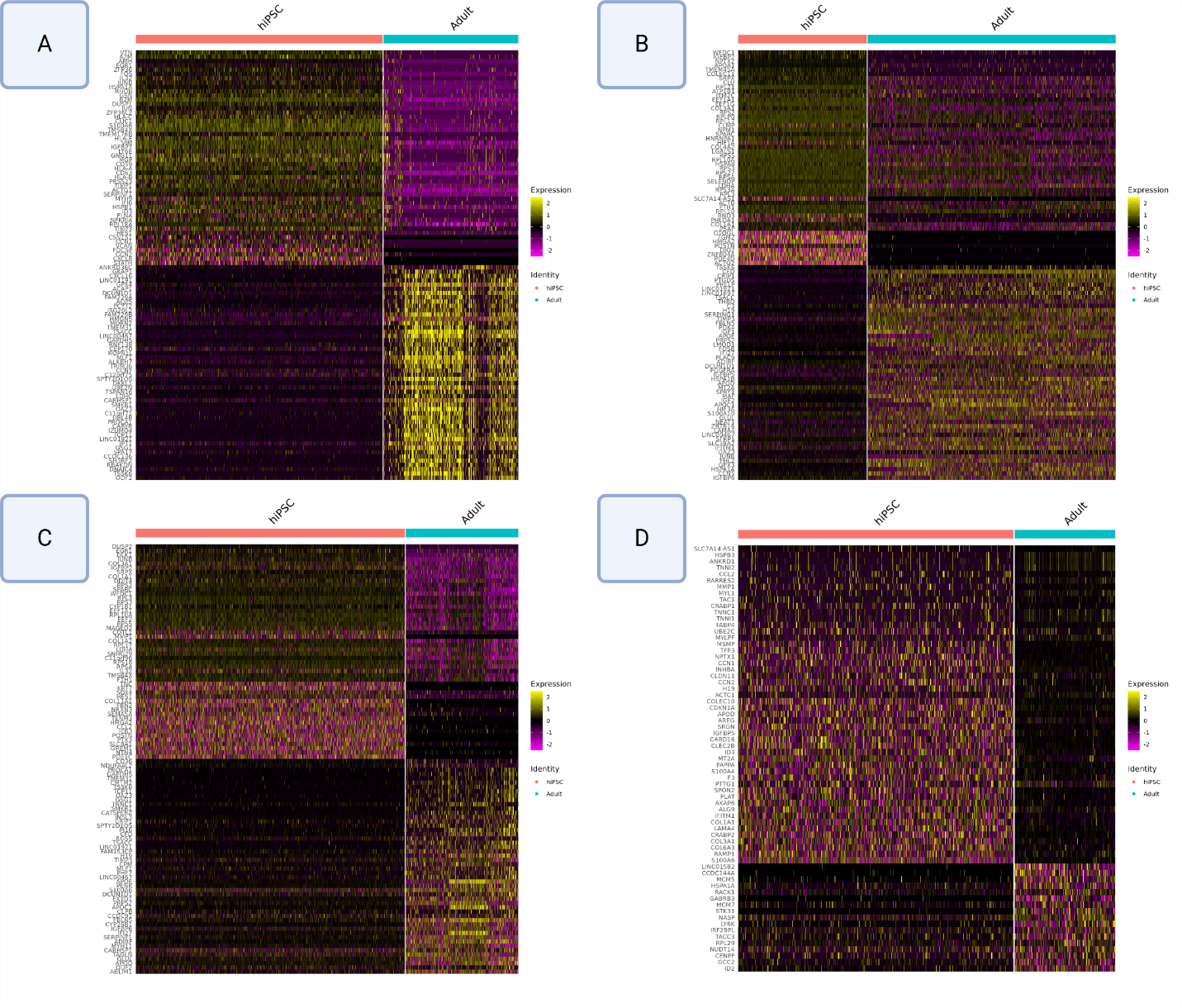


*Supporting file 3, Figure 3*. Heatmaps from the integrated datasets showing the DEGs between hiPSC-derived cell types and the corresponding adult cell type. A) Sertoli cells. B) Leydig cells. C) Peritubular myoid cells. D) SSCs.
